# Potential Risk Factors Associated with Human Cystic Echinococcosis: Systematic Review and Meta-analysis

**DOI:** 10.1371/journal.pntd.0005114

**Published:** 2016-11-07

**Authors:** Alessia Possenti, Raúl Manzano-Román, Carlos Sánchez-Ovejero, Belgees Boufana, Giuseppe La Torre, Mar Siles-Lucas, Adriano Casulli

**Affiliations:** 1 Department of Infectious, Parasitic and Immunomediated Diseases, Istituto Superiore di Sanità (ISS), Rome, Italy; 2 European Reference Laboratory for Parasites (EURLP), ISS, Rome, Italy; 3 Instituto de Recursos Naturales y Agrobiologia de Salamanca, IRNASA-CSIC, Salamanca, Spain; 4 World Health Organization Collaborating Centre for the epidemiology, detection and control of cystic and alveolar echinococcosis (in humans and animals), ISS, Rome, Italy; 5 Sapienza University of Rome, Department of Public health and Infectious Diseases, Rome, Italy; Universidad Nacional Autónoma de México, MEXICO

## Abstract

**Background:**

Scientific literature on cystic echinococcosis (CE) reporting data on risk factors is limited and to the best of our knowledge, no global evaluation of human CE risk factors has to date been performed. This systematic review (SR) summarizes available data on statistically relevant potential risk factors (PRFs) associated with human CE.

**Methodology/Principal Findings:**

Database searches identified 1,367 papers, of which thirty-seven were eligible for inclusion. Of these, eight and twenty-nine were case-control and cross-sectional studies, respectively. Among the eligible papers, twenty-one were included in the meta-analyses. Pooled odds ratio (OR) were used as a measure of effect and separately analysed for the two study designs. PRFs derived from case-control studies that were significantly associated with higher odds of outcome were “dog free to roam” (OR 5.23; 95% CI 2.45–11.14), “feeding dogs with viscera” (OR 4.69; 95% CI 3.02–7.29), “slaughter at home” (OR 4.67; 95% CI 2.02–10.78) or at “slaughterhouses” (OR 2.7; 95% CI 1.15–6.3), “dog ownership” (OR 3.54; 95% CI 1.27–9.85), “living in rural areas” (OR 1.83; 95% CI 1.16–2.9) and “low income” (OR 1.68; 95% CI 1.02–2.76). Statistically significant PRFs from cross-sectional studies with higher odds of outcome were “age >16 years” (OR 6.08; 95% CI 4.05–9.13), “living in rural areas” (OR 2.26; 95% CI 1.41–3.61), “being female” (OR 1.38; 95% CI 1.06–1.8) and “dog ownership” (OR 1.37; 95% CI 1.01–1.86).

**Conclusions/Significance:**

Living in endemic rural areas, in which free roaming dogs have access to offal and being a dog-owner, seem to be among the most significant PRFs for acquiring this parasitic infection. Results of data analysed here may contribute to our understanding of the PRFs for CE and may potentially be useful in planning community interventions aimed at controlling CE in endemic areas.

## Author Summary

Cystic echinococcosis (CE) is a chronic zoonotic disease causing serious global socio-economic losses in human and animal hosts. Two main aspects make it extremely difficult to study risk factors associated with human CE, the parasite’s unknown and apparently long incubation period which may last for several years, and the predominantly fecal-oral transmission route. This systematic review (SR) summarizes findings from relevant publications on this topic and provides a detailed list of potential risk factors (PRFs) associated with CE infection in humans. Free dog roaming, dogs having access to offal, being a dog-owner and slaughtering at home or using inadequately supervised slaughterhouses have all been shown to be highly statistically significant PRFs associated with the perpetuation of the parasite life cycle in endemic areas. The effect of other risk factors identified in this SR can vary between geographically different areas and societies and could reflect socio-cultural determinants of infection.

## Introduction

Cystic echinococcosis (CE), caused by the metacestode stage of the tapeworm *Echinococcus granulosus* sensu lato (s.l.), is a neglected zoonotic disease producing economic losses in animals and high morbidity and mortality rates in humans with huge health, social and economic consequences for communities affected [1, 2]. At global level, it has been estimated there are more than one million human CE cases with a disease burden between 1 and 3.6 million disability-adjusted life years (DALYS) [3, 4].

Humans become infected through the ingestion of *Echinococcus* spp. eggs that can develop into one or more fluid-filled cysts causing a chronic and life-threatening disease. In contrast to alveolar echinococcosis, CE may be considered as a chronic disabler rather than a killer. In fact, clinically diagnosed cases account for a small proportion of the total number of infected individuals who represent the major invisible portion of cases, leading to the underestimation and underreporting of CE.

The multi-host ecology and genotypic diversity of *E*. *granulosus* s.l. leads to complex host-parasite dynamics involving several intermediate (e.g., sheep, cattle, pigs and goats) and definitive (dogs, jackals and wolves) hosts. However, dogs are the major source of infection to humans, and the majority of documented human CE cases are caused by G1 genotype of *E*. *granulosus* sensu stricto (s.s.), in a life cycle that occurs mainly within a rural setting between sheep and shepherd dogs [5].

The geographical distribution and endemicity of CE differs by country and region and is influenced by different biotic and abiotic factors. Human infection in endemic regions also depends on a number of behavioral and socio-economic variables favoring close contact with parasite eggs [6]. Moreover, high environmental egg concentration in specific rural settings constitutes an epidemiologically important issue related to CE transmission [7].

The poorly understood and apparently long incubation period of this parasitic infection (which in most cases is lifelong), make it difficult to study risk factors associated with human CE. In addition, the fecal-oral route of transmission by direct oral uptake of *E*. *granulosus* s.l. eggs, through contact with dogs or contaminated matrices such as soil, water, and food, impede understanding the pathways of transmission [8]. In such terms, the study of CE etiology is extremely complex.

The objective of this research was to conduct a systematic review (SR) and meta-analyses of studies evaluating potential risk factors (PRFs) of CE using the Cochrane and PRISMA Group guidelines. This SR summarizes the findings of relevant publications on this topic, synthesizing the PRFs associated with CE infection in humans.

## Methods

### Review question and inclusion/exclusion criteria

The online search was carried out by combining keywords using Boolean operators AND/OR, “?” and “#”. The question mark (?), when used, expanded the search by looking for words with similar prefixes using more than one letter whereas the hash mark (#) expanded the search by looking for words with similar prefixes using one letter. The strategy developed in PubMed/Medline used queries for papers reporting abstracts on risk factors related to human CE. Thus, the final terms used for the search were “[echinococcus granulosus OR (echinococcus AND granulosus) OR e# granulosus OR cystic echinococcosis OR c# echinococcosis OR hydatidosis OR hydatid disease OR echinococcal] AND [risk factor# OR risk# OR exposure] AND [human# OR people OR person OR man OR men OR women OR woman OR patient# OR case# OR human population]”.

Primary research studies published or in press were considered eligible for inclusion. Other inclusion criteria based on study design were case-control, cross-sectional and cohort studies. Exclusion criteria included review articles, letters, editorials or opinion papers not containing primary data, duplicated data and studies on other echinococcosis causative agents (e.g. *Echinococcus multilocularis*).

### Study design: search strategy and data extraction

This SR and meta-analysis followed the Cochrane and PRISMA Group guidelines [9]. PRISMA check list is provided as supplementary material (S1 Checklist). The first online electronic search was conducted on the 20^th^ November 2014 and was updated on April 1^st^ 2016 in order to include recently published reports. The systematic search for abstracts/manuscripts was carried out by the Documentation Service for literature search at the Istituto Superiore di Sanità (Rome, Italy). The platform used for searching the databases was STN International–Fiz Karlsruhe (https://www.fiz-karlsruhe.de/). Principal data sources selected for the literature search included the following six bibliographic databases: the Medical Literature Analysis and Retrieval System Online (MEDLINE), Excerpta Medica Database (EMBASE), Science Citation Index (SciSearch), Biological Abstracts (BIOSIS), Centre for Agricultural Bioscience International (CABI) and Google Scholar. Duplicate articles were removed during the initial search. Later, article selection was based on title and abstract in relation to the keywords. Finally, full-text papers were screened for eligibility and data was extracted from selected studies by completing standardized Excel tables. Data reported in the extraction tables were as follows: paper identification (ID, sub-ID, first author, year of publication, title, journal, volume, page numbers), geographical area, country and year of study, study design (case-control, cross-sectional, cohort study), diagnostic method (ultrasonography, surgery, percutaneous techniques, X-ray, serology), PRFs and quality assessment. Data extraction was performed independently by two researchers (R M-R and C S-O); any disagreements were resolved either by consensus among researchers or arbitration by an additional independent researcher (M S-L) using standardized extraction forms to guarantee consistency and accuracy. Each article meeting the inclusion criteria was evaluated, and data relating to PRFs were extracted according to the following groups: association with dogs, slaughtering animals (at home or at slaughterhouses), gender, age, familial or ethnic clusters, living in rural areas, occupation, food/water contact, and socio-cultural level. Data from studies based only on serology were extracted, but due to the low accuracy of these diagnostic tests [10, 11], these studies were included in the meta-analysis only when ultrasonography detection was also reported. The literature search was restricted to 3 languages, English, Spanish and Italian but no date restriction was enforced. EndNote software was used for document management.

### Quality assessment

The quality of the studies included in this review was evaluated by two independent researchers using the Newcastle-Ottawa Scale (NOS) according to the Cochrane Handbook for Systematic Reviews [12, 13]. Studies were scored in two domains: selection of the study groups and exposure/outcome. A maximum score of 4 and 3 for each respective area was allocated out of a total possible score of 7. Study comparability was not assessed due to the absence of study controls for all risk factors.

### Research evidence: data- and meta-analysis

Statistical analysis was performed using the software Review Manager 5.2 (RevMan Version 5.2. Copenhagen: The Nordic Cochrane Centre, The Cochrane Collaboration, 2014; http://ims.cochrane.org/revman). Pooled odds ratio (OR) were used as a measure of effect and separately analysed for case-control and cross-sectional studies. Meta-analysis was conducted when at least two studies reported data on a single risk factor. The OR, with the relative 95% CI, was calculated for PRFs containing two or more studies and plotted using a forest plot. Cochran’s Q test was performed to assess the degree of heterogeneity between studies, and the I^2^ statistic was used to describe the percentage of total variation across studies as a result of heterogeneity. If the *p*-value of the Q test was <0.05 and I^2^ was >50%, heterogeneity was inferred, and the random-effect model was used. Otherwise, if heterogeneity was not detected, a fixed-effect model was adopted. Publication bias was quantified by inspection of funnel plots and computation of Egger [14] and Begg [15] probability values. A meta-regression analysis was conducted on each single risk factor reported in at least 3 studies. The following variables were taken into account, year of publication, total population, and quality scores. For each analysis, a linear regression model was built using the stepwise procedure (backward elimination) and results were presented as beta coefficients and *p*-values. The statistical significance was set at *p*<0.05. The meta-regression analysis was performed using SPSS for Windows, release 22.0 (BM Corp. Released 2013. IBM SPSS Statistics for Windows, Version 22.0. Armonk, NY: IBM Corp).

## Results

### Study selection process

**Fig 1:**
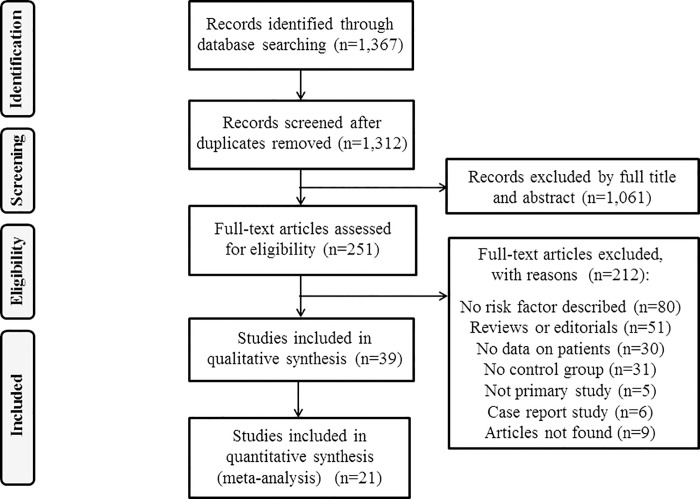
Searches performed and the number of articles returned and examined at each stage of the research on potential risk factors associated with human cystic echinococcosis (CE).

The literature search used in this study identified a total of 1,367 potentially relevant papers. Following an initial screening by title and abstract, 1,061 papers were excluded and 251 were retained for full text analysis (Fig 1). A second screening resulted in the exclusion of 212 papers based on the following criteria: no risk factor was reported, were not primary studies, had no data on patients, were reviews or editorials, had no control groups and other reasons (Fig 1; S2 Table). Data was extracted from a total of thirty-seven eligible papers (case-control studies n = 8; cross-sectional studies n = 29) (S1 Table). No cohort studies were identified. The geographical locations of the thirty-seven papers used in this review included Asia (n = 16), the Middle East (n = 8), South America (n = 6), Africa (n = 4), Europe (n = 2), and North America (n = 1). Papers used in the current SR were published between 1964 and 2014.

Of the cross-sectional studies, thirteen used ultrasonography as the reference method for CE detection, whereas only serology or ultrasonography and serology were used in the remaining sixteen papers. Meta-analyses were performed separately on cross-sectional studies reporting ultrasonography as the detection method and case-control studies using imaging techniques (ultrasonography and X-ray), or interventions (surgery and percutaneous techniques). Among the thirty-seven eligible papers, twenty-one (case-control studies n = 8; cross-sectional studies n = 13) were used for the meta-analyses (S1 Table). The assessment of the quality of the studies included in this meta-analysis was performed using NOS through the implementation of a ‘star system’. Of the 8 case control studies, 7 were allocated a 5-star rating and 1 study received a 3-star score. Within the cross-sectional studies, 6 and 7 star ratings were respectively assigned to 5 and 6 of these studies. The remaining two cross-sectional studies had a 3 and 4-star score respectively.

When studies were conducted using different diagnostic methods, or performed using different groups of individuals (for example adults versus children) at different time intervals (e.g. in different years or months) they were divided into sub-studies and each sub-study was analysed separately.

### Potential risk factors: meta-analysis using case-control studies

Fourteen risk factors were identified from case-control studies and meta-analysis was performed on eight of the included papers. Studies originated from Argentina (n = 1), Egypt (n = 1), Jordan (n = 1), Lebanon (n = 1), Peru (n = 1), Spain (n = 1), Turkey (n = 1) and Yemen (n = 1). These were hospital-based retrospective studies using control groups that were not affected by CE, recruited at hospital level and had similar demographic characteristics as those of the CE patients. Potential risk factors grouped in this meta-analysis were as follows: five were dog related (“dog free to roam”, “feeding dogs with viscera”, “having dog contact”, “dog ownership”, “dog dewormed infrequently or never”), three food- and water-borne related (“eating raw/unwashed vegetables”, “having a kitchen garden”, “drinking tap/piped water”), and six were socio-culturally related (“low income”, “low education”, “herding”, “slaughter at home” or at “slaughterhouses”, and “living in rural areas”). “Low education”, as described in the included sub-studies, was differentiated into primary (or lower) versus secondary education (or higher). The definition of “low-income” was based on direct socio-economic indicators such as receiving social and food aid from the state or indirect indicators such as not having a stone house or a telephone.

Seven PRFs were statistically significant (test for overall effect, *p*<0.05) with exposure associated with higher odds of outcome: “dog free to roam” (OR 5.23; 95% CI 2.45–11.14; *p*<0.0001), “feeding dogs with viscera” (OR 4.69; 95% CI 3.02–7.29; *p*<0.00001), “slaughter at home” (OR 4.67; 95% CI 2.02–10.78; *p*<0.0003) or at “slaughterhouses” (OR 2.7; 95% CI 1.15–6.3; *p*<0.02), “dog ownership” (OR 3.54; 95% CI 1.27–9.85; *p* = 0.02), “living in rural areas” (OR 1.83; 95% CI 1.16–2.9; *p*<0.01) and “low income” (OR 1.68; 95% CI 1.02–2.76; *p*<0.04).

Three PRFs increased the odds of infection but the results were not statistically significant: “dog contact” (OR 3.74; 95% CI 0.41–33.96; *p* = 0.24), “low education” (OR 1.39; 95% CI 0.89–2.16; *p* = 0.15) and “herding” (OR 1.33; 95% CI 0.8–2.21; *p* = 0.27).

For four PRFs, it was not possible to determine their effect on odds of infection: “dog dewormed infrequently or never” (OR 1.08; 95% CI 0.47–2.49; *p* = 0.86), “eating raw/unwashed vegetables” (OR 0.79; 95% CI 0.4–1.56; *p* = 0.5), “having a kitchen garden” (OR 0.61; 95% CI 0.18–2.09; *p* = 0.43) and “drinking tap/piped water” (OR 0.60; 95% CI 0.05–7.18; *p* = 0.68).

PRFs meta-analysed for case-control studies are summarized in Table 1. Forest plots, funnel plots and single weight of each publication contributing to the overall risk factors are presented in S1 Supplementary Information. With regards to the meta-regression analysis for case-control studies, the OR for “dog ownership” was inversely influenced by the quality score of the studies (beta = -0.98; *p* = 0.003).

**Table 1:** Cystic echinococcosis (CE) potential risk factors meta-analysed with related effect model used, odds ratio and confidence intervals (CI), significance of overall effect and numbers of papers included for case-control studies.

Potential risk factor	Effect model	Odds ratio [95% CI]	Overall effect	N° of sub-studies included
** Dog free to roam**	** (M-H, Fixed)**	** 5.23 [2.45–11.14]**	** p<0.0001**	** 2**
** Feeding dogs with viscera**	** (M-H, Fixed)**	** 4.69 [3.02–7.29]**	** p<0.00001**	** 4**
** Slaughter at home**	** (M-H, Fixed)**	** 4.67 [2.02–10.78]**	** p<0.0003**	** 2**
* Dog contact	(M-H, Random)	3.74 [0.41–33.96]	p = 0.24	2
** Dog ownership**	** (M-H, Random)**	** 3.54 [1.27–9.85]**	** p = 0.02**	** 5**
** Slaugtherhouses**	** (M-H, Fixed)**	** 2.70 [1.15–6.30]**	** p<0.02**	** 2**
** Living in rural areas**	** (M-H, Fixed)**	** 1.83 [1.16–2.90]**	** p<0.01**	** 2**
** Low income**	** (M-H, Fixed)**	** 1.68 [1.02–2.76]**	** p<0.04**	** 3**
* Low education	(M-H, Fixed)	1.39 [0.89–2.16]	P = 0.15	4
* Herding	(M-H, Random)	1.33 [0.80–2.21]	p = 0.27	8
§ Dog dewormed infrequently or never	(M-H, Fixed)	1.08 [0.47–2.49]	p = 0.86	2
§ Eating raw/unwashed vegetables	(M-H, Fixed)	0.79 [0.40–1.56]	P = 0.5	4
§ Having a kitchen garden	(M-H, Random)	0.61 [0.18–2.09]	p = 0.43	2
§ Drinking tap/piped water	(M-H, Random)	0.60 [0.05–7.18]	p = 0.68	4

Bold: significantly increase odds of infection

*: increase odds of infection but not significantly

§: no evidence of impact on infection risk.

### Potential risk factors: meta-analysis using cross-sectional studies

Eighteen PRFs were grouped from cross-sectional studies and meta-analysis was performed on thirteen of the twenty-nine included papers which originated from Argentina (n = 2), Canada (n = 1), China (n = 8), China and Mongolia (n = 1), Chile (n = 1), Greece (n = 1), India (n = 1), Iran (n = 5), Jordan (n = 1), Kyrgyzstan (n = 1), Libya (n = 1), Sudan (n = 1), Tunisia (n = 1), Turkey (n = 3) and Uruguay (n = 1). All these cross-sectional studies were community-based ultrasonography surveys. Potential risk factors evaluated in this meta-analysis were: two dog related (“dog ownership”, “feeding dogs with viscera”), five food- and water-borne related (“eating raw/unwashed vegetables”, “drinking well water”, “drinking spring water”, “drinking unboiled water”, “drinking tap/piped water”), two related to working activities (“livestock owner”, “being a farmer”), seven socio-culturally related (“slaughter at home”, “belonging to ethnic group Han”, “low income”, “low education”, “living in rural areas”) and five miscellaneous factors (“age >16 years”, “being female”, having “no knowledge on *Echinococcus*” and “family history of CE”). “Low education” was differentiated into primary (or lower) versus secondary level (or higher). “Low income” was defined based on socio-economic status as determined by being a recipient of government financial assistance or according to the profession of the head of the family.

Four PRFs were statistically significant (test for overall effect, *p*<0.05) with exposure associated with higher odds of outcome: “age >16 years” (OR 6.08; 95% CI 4.05–9.13; *p*<0.00001), “living in rural areas” (OR 2.26; 95% CI 1.41–3.61; *p*<0.0007), being female (OR 1.38; 95% CI 1.06–1.8; *p* = 0.02) and “dog ownership” (OR 1.37; 95% CI 1.01–1.86; *p* = 0.04).

Eight PRFs appeared to increase the odds of infection but results were not statistically significant. These were “belonging to ethnic group Han” (OR 2.19; 95% CI 0.66–7.26; *p* = 0.2), “being a farmer” (OR 2.18; 95% CI 0.66–7.22; p = 0.2), “feeding dogs with viscera” (OR 1.52; 95% CI 0.88–2.62; *p* = 0.13), “slaughter at home” (OR 1.19; 95% CI 0.94–1.5; *p* = 0.15), “drinking spring water” (OR 1.51; 95% CI 0.93–2.47; *p* = 0.1), “family history of CE” (OR 1.25; 95% CI 0.89–1.75; *p* = 0.2), “low income” (OR 1.45; 95% CI 0.72–2.91; *p* = 0.3) and “low education” (OR 3.12; 95% CI 0.19–51.32; *p* = 0.43).

For six PRFs, it was not clear whether they effected odds of infection: “eating raw/unwashed vegetables” (OR 1.13; 95% CI 0.63–2.05; *p* = 0.68), “drinking tap/piped water” (OR 1.07; 95% CI 0.63–1.81; *p* = 0.8), “livestock owner” (OR 0.99; 95% CI 0.7–1.4; *p* = 0.96), “drinking unboiled water” (OR 0.8; 95% CI 0.6–1.06; *p* = 0.12), “drinking well water” (OR 0.67; 95% CI 0.43–1.06; *p* = 0.08) and having “no knowledge on *Echinococcus*” (OR 0.23; 95% CI 0.05–1.07; *p* = 0.06). Table 2 shows the PRFs meta-analysed for cross-sectional studies. Forest plots, funnel plots and single weight of each publication contributing to the overall risk factors are presented in S2 Supplementary Information. Using meta-regression analysis, for cross-sectional studies, the OR for “belonging to ethnic group Han” was influenced directly by the quality score of the studies (beta = 2,672; *p* = 0.03) and inversely by the year of publication (beta = -2,139; *p* = 0.037).

**Table 2:** Cystic echinococcosis (CE) potential risk factors meta-analysed with related effect model used, odds ratio and confidence intervals (CI), significance of overall effect and numbers of papers included for cross-sectional studies.

Potential risk factor	Effect model	Odds ratio [95% CI]	Overall effect	N° of sub-studies included
** Age >16 years**	** (M-H, Fixed)**	** 6.08 [4.05–9.13]**	** p<0.00001**	** 4**
* Low education	(M-H, Random)	3.12 [0.19–51.32]	p = 0.43	2
** Living in rural areas**	** (M-H, Fixed)**	** 2.26 [1.41–3.61]**	** p<0.0007**	** 3**
* Ethnic group: Han	(M-H, Random)	2.19 [0.66–7.26]	p = 0.2	4
* Being a farmer	(M-H, Random)	2.18 [0.66–7.22]	p = 0.2	3
* Feeding dogs with viscera	(M-H, Fixed)	1.52 [0.88–2.62]	p = 0.13	3
* Drinking spring water	(M-H, Fixed)	1.51 [0.93–2.47]	p = 0.1	2
* Low income	(M-H, Fixed)	1.45 [0.72–2.91]	p = 0.3	2
** Being female**	** (M-H, Random)**	** 1.38 [1.06–1.80]**	** p = 0.02**	** 7**
** Dog ownership**	** (M-H, Random)**	** 1.37 [1.01–1.86]**	** p = 0.04**	** 8**
* Family history of CE	(M-H, Fixed)	1.25 [0.89–1.75]	p = 0.2	2
* Slaughter at home	(M-H, Fixed)	1.19 [0.94–1.50]	p = 0.15	4
§ Eating raw/unwashed vegetables	(M-H, Fixed)	1.13 [0.63–2.05]	p = 0.68	2
§ Drinking tap/piped water	(M-H, Fixed)	1.07 [0.63–1.81]	p = 0.8	3
§ Livestock owner	(M-H, Fixed)	0.99 [0.70–1.40]	p = 0.96	5
§ Drinking unboiled water	(M-H, Fixed)	0.80 [0.60–1.06]	p = 0.12	2
§ Drinking well water	(M-H, Fixed)	0.67 [0.43–1.06]	p = 0.08	3
§ No knowledge on *Echinococcus*	(M-H, Random)	0.23 [0.05–1.07]	p = 0.06	2

**Bold**: significantly increase odds of infection

*: increase odds of infection but not significantly

§: no evidence of impact on potential infection risk.

## Discussion

### Case-control studies

**Fig 2:**
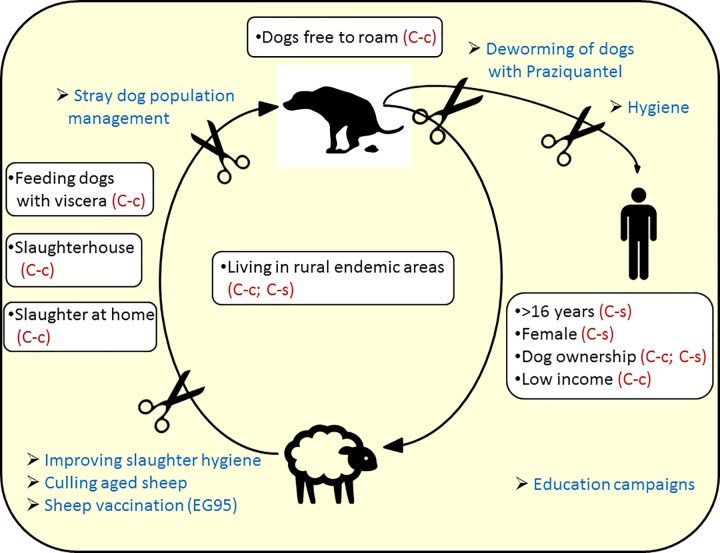
Key findings on the highly statistically significant (p<0.01) potential risk factors associated with human cystic echinococcosis (CE) derived from the meta-analysis of case-control (C-c) and cross-sectional (C-s) studies. In blue: possible vertical and horizontal interventions aimed at decreasing or interrupting the transmission of CE to humans.

For case-control studies, it was interesting to note that PRFs dealing with the perpetuation of the parasite life cycle between dogs and sheep are among the most statistically significant risk factors highlighted in this SR (test for overall effect, *p*<0.01). In fact, living in endemic rural areas, in which free roaming dogs have access to offal and being a dog owner, seem to be the most highly significant PRFs for acquiring this parasitic infection (Fig 2). These PRFs such as “dog free to roam” (I^2^ = 24%), “feeding dogs with viscera” (I^2^ = 32%), “slaughter at home (I^2^ = 32%), at “slaughterhouses” (I^2^ = 29%), “living in rural areas” (I^2^ = 45%) and “low income” (I^2^ = 0) were shown in this SR to have significantly higher odds of infection and demonstrated a low degree of heterogeneity between studies (S1 Supplementary Information). In contrast, although a similarly higher risk of infection was observed for “dog ownership”, a higher degree of heterogeneity (I^2^ = 77%) was reported. It is interesting to note that not only “slaughter at home” but also the use of “slaughterhouses” seemed to be a proxy for acquiring this disease. This suggests that in areas where the studies were conducted (Peru and Argentina) [16, 17], dogs had access to infected offal due to the mismanagement of infected organ disposal, thus increasing the probability of CE transmission to humans [18]. Health policy strategies such as mandatory disease notification at slaughterhouses, and/or sanitation may help to define directed interventions in order to interrupt CE transmission. In this respect, collecting epidemiological data at slaughterhouses on infected sheep (as the most important intermediate host), such as age class (young versus adults) and geographical origin, may contribute towards improving surveillance of CE [16, 19].

Potential risk factors increasing the odds of acquiring CE infection but having a weak and non-statistically significant association such as “dog contact” (I^2^ = 78%), “low education” (I^2^ = 0) and “herding” (I^2^ = 62%), demonstrated variable heterogeneity between studies. Contrary to “dog ownership”, for which a strong association was detected, “dog contact” seemed to present a lower probability of exposure to infection. However, socio-cultural factors that influence degree of dog contact such as "low education” and “herding” may represent confounding factors.

For the remaining PRFs such as “dogs dewormed infrequently or never” (I^2^ = 46%), “eating raw/unwashed vegetables” (I^2^ = 0), “having a kitchen garden” (I^2^ = 74%) and “drinking tap/piped water” (I^2^ = 89%) for which the statistical association was weak and non-significant, the heterogeneity of studies was variable. Results from case-control studies analysed in this SR do not provide significant evidence to indicate that CE is a strictly food- or water-borne disease.

### Cross-sectional studies

Regarding cross-sectional studies, the identified associations were more difficult to interpret due to the potential selection bias typically introduced by this study design, especially regarding age and gender.

Among the PRFs showing statistically significant evidence of increasing odds of CE infection, the heterogeneity of the studies were quite high for “age >16 years” (I^2^ = 55%), “living in rural areas” (I^2^ = 60%), “being female” (I^2^ = 62%) and “dog ownership” (I^2^ = 67%). Among potential confounding factors, “age > 16” may be linked to the chronic course of this parasitic disease that may remain asymptomatic for years. In fact, CE can be detected by chance years after the initial infection, for instance because the probability of being examined by ultrasound increases with age. With regards to “being female”, although a potential confounding factor, some activities executed by women in rural endemic areas, such as feeding and handling of dogs, could also reflect a higher exposure to the parasite.

Results regarding the PRFs which increase the odds of infections with a weak and non-significant association such as “belonging to ethnic group Han” (I^2^ = 87%), “being a farmer” (I^2^ = 78%), “feeding dogs with viscera” (I^2^ = 0), “drinking spring water (I^2^ = 0), “low income” (I^2^ = 41%) and “low education” (I^2^ = 82%), the heterogeneity of studies was variable. These PRFs showed no statistically significant association and may be regarded as socio-economic determinants which could potentially influence people’s exposure and vulnerability to non-communicable diseases and may be considered both drivers as well as confounding factors [20].

The PRFs for which there was no evidence of an impact on CE infection risk and no significant association, mostly demonstrated high variability in heterogeneity of studies such as “eating raw/unwashed vegetables” (I^2^ = 44%), “drinking tap/piped water” (I^2^ = 0), “livestock owner” (I^2^ = 0), “drinking unboiled water” (I^2^ = 64%), “drinking well water” (I^2^ = 0%) and having “no knowledge on *Echinococcus*” (I^2^ = 94%). Similar to case-control studies, PRFs from cross-sectional studies directly linked to food- and water-borne pathways of transmission do not appear to impact significantly on infection risk for CE. In fact, with the exception of “drinking spring water”, that could represent a third variable as a confounding factor, this SR has shown that the risk of CE transmission through the ingestion of food and water contaminated with *E*. *granulosus* s.l. eggs was not evidence based and is potentially anecdotal.

### Limits in observational studies

Observational studies (such as case-controls and cross-sectional) have intrinsic limits and advantages that should be taken into account during the evaluation of PRFs for acquiring CE. For instance, case-control studies are cost effective and efficient in the study of rare diseases with long latency periods such as CE, but they are particularly prone to selection, recall and observational bias. Moreover, the temporal sequence between exposure and disease may be difficult to determine in these studies [21].

On the other hand, cross-sectional studies are relatively quick and easy to conduct and are good for descriptive analyses and for generating hypotheses [21]. They also provide estimates of prevalence for all measured factors which is important for assessing the burden of disease in specific settings, such as rural areas for CE, and in planning and allocating health resources. However, when conducting cross-sectional studies it is difficult to determine whether the outcome (diseased or healthy) followed exposure (to a particular risk factor) in time [22]. In particular, cross-sectional studies on CE can be biased because of the potential presence of false “non-exposed” groups for a specific risk factor. For instance, some human protective behaviors such as “not slaughtering at home” are not determining exposure in some specific cases because neighbors may be “slaughtering at home” thus contributing to the maintenance of the life cycle in this particular setting.

It is noteworthy that for certain PRFs, such as “age” and “gender”, a spurious association can be present between a given PRF and CE, as a result of the influence of other confounding variables. For instance, “age” could be considered a confounder because CE is asymptomatic or paucisymptomatic for years, thus the probability of detecting the disease increases with age. Similarly, women are usually numerically more represented during ultrasonography screening than men, which increases the possibility of having a gender selection bias during sampling. In this sense, some of the PRFs reported above may represent potential confounders introducing bias in observational studies. Unfortunately, in the current meta-analysis only aggregated data were available both for case-control and cross-sectional studies, thus the confounding effect on PRFs due to other variables could not be adjusted.

In addition, enforcing a language constraint on the literature search such as that used in this study, may have restricted the retrieval of material published in other languages. However, it is widely perceived that relevant peer reviewed studies are published in English, for example even though Chinese was excluded from the language search, the highest number of retrieved studies used for data extraction and meta-analysis had originated from China (n = 9). Furthermore, a recent study on the effect of language restriction on systematic review-based meta-analyses in conventional medicine found no evidence of bias as a result of language restriction [23]. All these arguments may be considered as limiting factors for the interpretation of PRFs linked to a disease with a long latency period such as CE.

This meta-analysis identified a number of PRFs that were statistically associated with higher odds of acquiring CE infection. Although control measures adopted by several countries to interrupt the life cycle of *E*. *granulosus* s.l. may in theory have an impact on PRFs, we have not been able to evaluate the effect of control measures on PRFs in this meta-analysis. This is largely due to the fact that the cross-sectional studies included in this SR were epidemiological surveys or risk factor assessments generating baseline CE data using ultrasonography and questionnaires. Such studies are usually a pre-requisite for the implementation of control programs and there is no known association between baseline data generated using ultrasonography (which represents a snapshot of the epidemiological situation of previous years) and control programmes that take decades of sustained effort to be effective.

### Pathways of infection and interventions

Several PRFs with relatively high odds ratio reported in this SR may explain how dogs can acquire echinococcosis (e.g., “dog free to roam”, “feeding dogs with viscera”, “slaughter at home) but are not able to elucidate the main pathways of CE transmission to humans. Although these pathways remain unclear, the majority of the PRFs associated with CE are related to dogs which probably represent the most important source of infection for humans [24]. Infection can occur either directly through close contact with dogs or indirectly through the ingestion of eggs present in and/or on contaminated matrices with human behavior and hygiene practices being essential for the fecal-oral pathways of transmission [7, 8]. A number of specific and oriented interventions aimed at decreasing or interrupting the probability of CE transmission to humans are depicted in Fig 2 [24].

Results of this SR seem to suggest that the direct or indirect contamination of hands with *E*. *granulosus* s.l. eggs excreted by dogs appears to represent one of the most important pathways of transmission for human CE as compared to egg ingestion through contaminated food and water. It is reasonable to hypothesize that human infection in areas with high infection rates in dogs would be driven mainly independently of food and water. However, assessing the real infection risks for human CE cannot be determined through SRs on PRFs. The greater majority of questionnaires relevant to the determination of risk factors associated with CE transmission have concentrated on information related to dog definitive hosts (e.g., type of food they are feed, whether free to roam, dog contact, dog ownership etc). Although a few studies have investigated the occurrence of eggs in soil samples from endemic areas [7, 8], to the best of our knowledge, no studies have thoroughly assessed the extent of contamination in and around human settlements with viable *Echinococcus* spp. eggs and how that would drive human CE infection. The implementation of specifically designed questionnaires and tailored molecular-epidemiological studies sampling different matrices (such as soil, fomites, water, vegetables etc) will assist in understanding factors affecting CE exposure in specific endemic settings and consequently the pathways of transmission in more detail.

In addition, some pathways of transmission identified in this SR can vary between geographically different areas and societies and could reflect socio-cultural determinants of infection, for example “belonging to ethnic group Han”. Additionally, a number of socio-cultural determinants highlighted in this SR, such as “dog dewormed infrequently or never” and having “no knowledge on *Echinococcus*” showed a weak non-significant statistical association with odds of infection. Thus, understanding differences in single socio-cultural determinants could be relevant also for implementing interventions aimed at decreasing or interrupting the transmission of CE.

Surprisingly, two of the main integrated strategies usually applied in control campaigns against CE (education and dog deworming) showed no clear statistical relationship with human infection in this SR. In fact, with the exception of the Icelandic hydatid campaign, health education for prevention of CE on its own has shown little influence in the reduction of *E*. *granulosus* transmission [25], although it may be crucial in control programs to allow people to understand how CE transmission can be interrupted. Regarding deworming of dogs, effectiveness of this intervention depends on the frequency of drug administration and consequently the variability of timing with regards to dosing may have influenced the statistical significance observed here for this PRF [24]. In addition, the incorporation of the use of the EG95 vaccine in control programmes, which has been shown to protect sheep against *E*. *granulosus* infection [26], could be useful in this setting, especially for reducing the duration of interventions [27].

In general, interventions aimed at mitigating or interrupting the transmission of CE mainly focus on improvement of hygiene at abattoirs, implementing education campaigns and primary health care, deworming of dogs with praziquantel, vaccination of sheep and culling of aged sheep [28, 29, 30]. Some of these approaches were successful when implemented on a large scale or on an island-based model. Low impact on CE control was achieved using small scale or continental-based interventions, with no feasible border control which subsequently led to parasite spillover from neighboring infected areas. For a detailed review of control campaigns and a critical discussion on varying degrees of success the reader is referred to a comprehensive report on this topic [25].

Parasitic infections including CE are typically associated with poor and often marginalized communities. Most interventions on CE are tailored to indirectly decrease the burden of CE in humans through vertical interventions in animals [31]. Primary health interventions aiming at directly decreasing the burden in humans are to be encouraged. In fact, these actions should target the population affected by CE through active search for carriers using ultrasound surveys. Such interventions are aimed to allocate people to treatment and generate baseline data for risk calculation and cost/benefit analyses. Thus, extensive ultrasound screening in endemic rural areas to mitigate sampling bias introduced through self-enrollment in cross-sectional studies is highly recommended.

## Supporting Information

S1 Checklist PRISMA checklist reporting items for this Systematic Review and Meta-Analyses. (DOC)

S1 Table List of studies included in the systematic review after full text screening. (PDF)

S2 Table List of studies excluded from the systematic review after full text screening. (PDF)

S1 Supplementary Information Case-control studies reporting forest and funnel plot analysis on single potential risk factors. (PDF)

S2 Supplementary Information Cross-sectional studies reporting forest and funnel plot analysis on single potential risk factors. (PDF)

S1 Flow Diagram PRISMA flow diagram. (DOC)
