# Supplementary material for: Potential Risk Factors Associated with Human Cystic Echinococcosis: Systematic Review and Meta-analysis
Source: PLoS Negl Trop Dis. 2016 Nov 7;10(11):e0005114. doi: 10.1371/journal.pntd.0005114 (PMC5098738; doi:10.1371/journal.pntd.0005114)
Supplement: S1 Table — (PDF) [file pntd.0005114.s002.pdf]

Supplementary Table 1. List of studies included in the systematic review after full text screening.

TITLE	REFERENCE	AUTHOR'S NAME	YEAR	STUDY DESIGN	DIAGNOSTIC METHOD
**Epidemiology of echinococcosis in the middle east. 3. A. Study of hydatid disease patients from the city of Beirut**	**The American Journal of Tropical Medicine and Hygiene. 13, 681-5.**	**K. Abou-Daoud, et al.**	**1964**	**Case control**	**surgery**
Prevalence and risk factors for echinococcal infection in a rural area of northern Chile: a household-based cross-sectional study	PLOS Neglected Tropical Diseases. 8, 8, e3090.	G. Acosta-Jamett, et al.	2014	Cross-sectional	serology
Seroprevalence of human cystic echinococcosis and risk factors in animal breeders in rural communities in Denizli, Turkey	The Journal of Infection in Developing Countries. 8, 9, 1188-94.	S. Akalin, et al.	2014	Cross-sectional	serology
**HLA class II alleles: susceptibility or resistance to cystic echinococcosis in Yemeni patients**	**Parasitology Research. 107, 2, 355-61.**	**A. B. Al-Ghoury, et al.**	**2010**	**Case control**	**not reported**
Seroepidemiology of Human Hydatidosis Using AgB-ELISA Test in Arak, Central Iran	Iranian Journal of Public Health. 42, 4, 391-6.	M. Asghari, et al.	2013	Cross-sectional	serology
**The evaluation of HLA-DRB1 antigens as susceptibility markers for unilocular cystic echinococcosis in Egyptian patients**	**Parasitology Research. 92, 6, 473-7.**	**M. E. Azab, et al.**	**2004**	**Case control**	**surgery, imaging and serology**
Seroepidemiology of Human Hydatidosis in Golestan Province, Iran	Iranian J Parasitology. 2, 2, 20-24.	M Baharsefat, et al.	2007	Cross-sectional	serology
An epidemiological survey of cystic echinococcosis among Tibetan school pupils in West China	Annals of Tropical Paediatrics. 21, 3, 235-8.	Y. Bai, et al.	2001	Cross-sectional	serology
**Survey on cystic echinococcosis in Tibetans, West China**	**Acta Tropica. 82, 3, 381-5.**	**Y. Bai, et al.**	**2002**	**Cross-sectional**	**ultrasound** and serology

**Echotomographic and serological population-based study of hydatidosis in central Tunisia**	**Acta Tropica. 49, 2, 149-53.**	**A. Bchir, et al.**	**1991**	**Cross-sectional**	**ultrasound**
**A community-based study to examine the epidemiology of human cystic echinococcosis in Rio Negro Province, Argentina**	**Acta Tropica. 136, 81–88.**	**G. M. Bingham, et al.**	**2014**	**Cross-sectional**	**ultrasound**
**Risk factors for Echinococcus granulosus infection: a case-control study**	**The American Journal of Tropical Medicine and Hygiene. 62, 3, 329-34.**	**A. Campos-Bueno, et al.**	**2000**	**Case control**	**Surgery, imaging and serology**
**Risk factors associated with human cystic echinococcosis in Florida, Uruguay: results of a mass screening study using ultrasound and serology**	**The American Journal of Tropical Medicine and Hygiene. 58, 5, 599-605.**	**C. Carmona, et al.**	**1998**	**Cross-sectional**	**ultrasound**
A sero-epidemiologic study on cystic echinococcosis in midwestern region of Turkey	Saudi Medical Journal. 26, 2, 350-1.	Z. Cetinkaya, et al.	2005	Cross-sectional	serology
**Risk factors associated with human cystic echinococcosis in Jordan: results of a case-control study**	**Annals of Tropical Medicine and Parasitology. 94, 1, 69-75.**	**P. M. Dowling, et al.**	**2000**	**Case control**	**surgery**
Seroprevalence of IgG antibodies against Echinococcus granulosus in the population of the region of Thessaly, Central Greece	PLoS One. 7, 5, e37112.	V. Fotiou, et al.	2012	Cross-sectional	serology
Cystic echinococcosis on the northeast of the provincial Reserve of Pampa de Achala, Córdoba, Argentina	Revista de Medicina Veterinaria. 89, 2, 41-44.	J. C. González Peralta, et al.	2008	Cross-sectional	serology
Seroprevalence of parasitic zoonoses and their relationship with social factors among the Canadian Inuit in Arctic regions	Diagnostic Microbiology and Infectious Disease. 78, 4, 404-10.	S. Goyette, et al.	2014	Cross-sectional	serology
Sonographical and serological survey of human cystic echinococcosis and analysis of risk factors associated with seroconversion in rural	Zoonoses Public Health. 58, 8, 582-8.	M. F. Harandi, et al.	2011	Cross-sectional	serology

communities of Kerman, Iran					
**TAP1 and TAP2 gene polymorphisms in childhood cystic echinococcosis**	**Parasitology International. 59, 2, 283-5.**	**N. Kiper, et al.**	**2010**	**Case control**	**imaging and serology**
**A case-control study of the risk factors for cystic echinococcosis among the children of Rio Negro province, Argentina**	**Annals of Tropical Medicine and Public Health. 96, 1, 43-52.**	**E. J. Larrieu, et al.**	**2002**	**Case control**	**ultrasound**
**Identification of risk factors for cystic echinococcosis in a peri-urban population of Peru**	**Transactions of the Royal Society of Tropical Medicine and Hygiene. 102, 1, 75-8.**	**P. L. Moro, et al.**	**2008**	**Case control**	**surgery**
**A province-based study using sampling method to investigate the prevalence of cystic echinococcosis among primary school children in Manisa, Turkey**	**Acta Tropica. 103, 2, 116-22**	**U. Z. Ok, et al.**	**2007**	**Cross-sectional**	**ultrasound**
The seroprevalences of cystic echinococcosis, and the associated risk factors, in rural-agricultural, bedouin and semi-bedouin communities in Jordan	Annals of Tropical Medicine and Public Health. 97, 5, 511-20.	A. M. Qaqish, et al.	2003	Cross-sectional	serology
Human cystic echinococcosis in nomads of south-west Islamic Republic of Iran	Eastern Mediterranean Health Journal. 13, 1, 41-8.	A. Rafiei, et al.	2007	Cross-sectional	serology
Echinococcosis on the Tibetan Plateau: prevalence and risk factors for cystic and alveolar echinococcosis in Tibetan populations in Qinghai Province, China	Parasitology. 127 Suppl, S109-20.	P. M. Schantz, et al.	2003	Cross-sectional	X-ray or ultrasound plus serology
**An extensive ultrasound and serologic study to investigate the prevalence of human cystic echinococcosis in northern Libya**	**The American Journal of Tropical Medicine and Hygiene. 60, 3, 462-8.**	**M. A. Shambesh, et al.**	**1999**	**Cross-sectional**	**ultrasound** and serology

Human hydatidosis : an under discussed occupational zoonosis in India	Helminthologia. Vol. 50, No. 2, pp. 87-90.	B. B. R. S. Singh et al.	2013	Cross-sectional	serology
**Cystic echinococcosis in Mundari tribe-members of South Sudan**	**Pathogen Global Health. 107, 6, 293-8.**	**B. T. Stewart, et al.**	**2013**	**Cross-sectional**	**ultrasound**
**Echinococcosis in Tibetan populations, western Sichuan Province, China**	**Emerging Infectious Diseases journal. 11, 12, 1866-73.**	**L. Tiaoying, et al.**	**2005**	**Cross-sectional**	**ultrasound**
**Human cystic echinococcosis in Kyrgystan: an epidemiological study**	**Acta Tropica. 85, 1, 51-61**	**P. R. Torgerson, et al.**	**2003**	**Cross-sectional**	**ultrasound**
Investigation of risk factors for development of human hydatidosis among households raising livestock in Tibetan areas of western Sichuan province	Chinese journal of parasitology & parasitic disease. 19, 2, 93-6.	Q. Wang, et al.	2001	Cross-sectional	serology
**Cystic echinococcosis in semi-nomadic pastoral communities in north-west China**	**Transactions of the Royal Society of Tropical Medicine and Hygiene. 95, 2, 153-8.**	**Y. H. Wang, et al.**	**2001**	**Cross-sectional**	**ultrasound**
**Human cystic echinococcosis in two Mongolian communities in Hobukesar (China) and Bulgan (Mongolia)**	**Transactions of the Royal Society of Tropical Medicine and Hygiene. 99, 9, 692-8.**	**Y. Wang, et al.**	**2005**	**Cross-sectional**	**ultrasound**
**Community surveys and risk factor analysis of human alveolar and cystic echinococcosis in Ningxia Hui Autonomous Region, China**	**Bulletin of the World Health Organization. 84:714-721.**	**Y. R. Yang, et al.**	**2006**	**Cross-sectional**	**ultrasound** and serology
**Cystic and alveolar echinococcosis: an epidemiological survey in a Tibetan population in southeast Qinghai, China**	**Japanese journal of infectious diseases. 61, 242-246.**	**S. H. Yu, et al.**	**2008**	**Cross-sectional**	**ultrasound**
The serological study of cystic echinococcosis and assessment of surgical cases during 5 years (2007-2011) in Khorram Abad, Iran	Nigerian Journal of Clinical Practice. 16, 2, 221-5.	M. Zibaei, et al.	2013	Cross-sectional	serology

In **bold,** studies used for meta-analysis
