# Supplementary material for: Potential Risk Factors Associated with Human Cystic Echinococcosis: Systematic Review and Meta-analysis
Source: PLoS Negl Trop Dis. 2016 Nov 7;10(11):e0005114. doi: 10.1371/journal.pntd.0005114 (PMC5098738; doi:10.1371/journal.pntd.0005114)
Supplement: S2 Table — (PDF) [file pntd.0005114.s003.pdf]

Supplementary Table 2. List of studies excluded from the systematic review after full text screening.

ID	Author's Name	Reference	Article Title	Reason for excluding
**1**	C. M. Budke, et al.	Vet Parasitol, 127, 1, 43-49	A canine purgation study and risk factor analysis for echinococcosis in a high endemic region of the Tibetan plateau	NO DATA ON PATIENTS
**2**	E. V. Noormahomed, et al.	PLoS Negl Trop Dis, 8, 9, e3121	A cross-sectional serological study of cysticercosis, schistosomiasis, toxocariasis and echinococcosis in HIV-1 infected people in Beira, Mozambique	NO CONTROL GROUP
**3**	P. M. Dowling and P. R. Torgerson	Ann Trop Med Parasitol, 94, 3, 241-5	A cross-sectional survey to analyse the risk factors associated with human cystic echinococcosis in an endemic area of mid-Wales	NO RISK FACTORS DESCRIBED
**4**	J. A. O'Hern and L. Cooley	Med J Aust, 199, 2, 117-20	A description of human hydatid disease in Tasmania in the post-eradication era	NO RISK FACTORS DESCRIBED
**5**	R. W. Roundy	Soc Sci Med, 12, 2d, 121-30	A model for combining human behavior and disease ecology to assess disease hazard in a community: rural Ethiopia as a model	NO DATA ON PATIENTS
**6**	U. Bakal, et al.	Acta Trop, 123, 2, 91-5	A portable ultrasound based screening study on the prevalence and risk factors of cystic echinococcosis in primary school children in East Turkey	NO RISK FACTORS DESCRIBED
**7**	N. I. Ramadan and S. I. el Damaty	J Egypt Soc Parasitol, 30, 1, 329-39	A preliminary screening study on human cystic echinococcosis in Cairo slaughter house personnel	NO DATA ON PATIENTS
**8**	M. Conchedda, et al.	Parasitol Int, 59, 3, 454-9	A retrospective analysis of human cystic echinococcosis in Sardinia (Italy), an endemic Mediterranean region, from 2001 to 2005	NO RISK FACTORS DESCRIBED
**9**	G. Ivanov	Ann Trop Med Parasitol, 90, 2, 167-71	A study of pulmonary hydatid disease in children. 1. Epidemiological and clinical characteristics	NO RISK FACTORS DESCRIBED
**10**	B. Otero-Abad and P. R.	PLoS Negl Trop Dis, 7, 6,	A systematic review of the epidemiology of echinococcosis in	REVIEW

	Torgerson	e2249	domestic and wild animals	
**11**	C. M. Budke, et al.	Am J Trop Med Hyg, 88, 6, 1011-27	A systematic review of the literature on cystic echinococcosis frequency worldwide and its associated clinical manifestations	REVIEW
**12**	H. H. Hu, et al.	Epidemiol Infect, 142, 6, 1214-20	A village-based multidisciplinary study on factors affecting the intensity of cystic echinococcosis in an endemic region of the Tibetan plateau, China	NO DATA ON PATIENTS
**13**	D. Waltner-Toews, et al.	EcoHealth, 2, 2, 155-164	Agro-urban Ecosystem Health Assessment in Kathmandu, Nepal: Epidemiology, Systems, Narratives	NO CONTROL GROUP
**14**	E. Perez-Rodriguez, et al.	Rev Clin Esp, 195, 3, 138-40	An epidemiological study on the relations of patients with hydatid disease. A high-risk population?	NO RISK FACTORS DESCRIBED
**15**	M. Pappaioanou, et al.	Am J Trop Med Hyg, 26, 4, 732-42	An evolving pattern of human hydatid disease transmission in the United States	NO CONTROL GROUP
**16**	C. N. Macpherson, et al.	Parasitology, 127 Suppl, S21-35	Application of ultrasound in diagnosis, treatment, epidemiology, public health and control of Echinococcus granulosus and E. multilocularis	NO RISK FACTORS DESCRIBED
**17**	M. E. Azab, et al.	J Egypt Soc Parasitol, 34, 1, 183-96	Association of some HLA-DRB1 antigens with Echinococcus granulosus specific humoral immune response	NOT FOUND ARTICLE
**18**	J. Richter, et al.	Euro Surveill, 14, 22,	Autochthonous cystic echinococcosis in patients who grew up in Germany	NO CONTROL GROUP
**19**	J. Chen, et al.	Parasit Vectors, 5, 152	Canine and feline parasitic zoonoses in China	NO DATA ON PATIENTS
**20**	I. Buishi, et al.	Ann Trop Med Parasitol, 100, 7, 601-10	Canine echinococcosis in Turkana (north-western Kenya): a coproantigen survey in the previous hydatid-control area and an analysis of risk factors	NO DATA ON PATIENTS
**21**	M. G. Verso, et al.	Med Lav, 91, 2, 135-41	Case study of a population of subjects in Sicily operated for hydatidosis: correlation with profession and living environment	NO RISK FACTORS DESCRIBED
**22**	L. A. Al-Shibani, et al.	Trop Biomed, 29, 1, 18-23	Cases of hydatidosis in patients referred to Governmental hospitals for cyst removal in Sana'a City, Republic of Yemen	NO RISK FACTORS DESCRIBED
**23**	X. X. Li and X. N. Zhou	Parasit Vectors, 6, 79	Co-infection of tuberculosis and parasitic diseases in humans: a systematic review	REVIEW

**24**	W. B. Zhang, et al.	Chin Med J (Engl), 124, 19, 3176-9	Community survey, treatment and long-term follow-up for human cystic echinococcosis in northwest China	NO RISK FACTORS DESCRIBED
**25**	O. K. Aribas, et al.	Eur J Cardiothorac Surg, 21, 3, 489-96	Comparison between pulmonary and hepatopulmonary hydatidosis	NO RISK FACTORS DESCRIBED
**26**	A. ITO, et al.	Parasitology, 140, Special Issue 13, 1547-1550	Control of cestode zoonoses in Asia: role of basic and applied science	REVIEW
**27**	A. Ito, et al.	Acta Trop, 86, 1, 3-17	Control of echinococcosis and cysticercosis: a public health challenge to international cooperation in China	REVIEW
**28**	R. Flores Castro	Gac Med Mex, 146, 6, 423-9	Current situation of the most frequent zoonosis in the world	REVIEW
**29**	A. Ito, et al.	PLoS Negl Trop Dis, 8, 6, e2937	Cystic echinococcoses in Mongolia: molecular identification, serology and risk factors	NO RISK FACTORS DESCRIBED
**30**	B. B. Alessandra Siracusano, Federica Delunardo, Paola Margutti, and E. P. e. R. R. Elena Ortona	Not Ist Super Sanità, 17, 2, 3-6	Cystic echinococcosis : a health problem that calls for a multidisciplinary approach	REVIEW
**31**	Edmundo Juan Larrieu and M. G. Cacheau	Salud(i)Ciencia, 17, 7, 627-632	Cystic echinococcosis epidemiological surveillance: modern strategies and technologies	REVIEW
**32**	P. S. Craig, et al.	Pathog Glob Health, 106, 6, 373-5	Cystic echinococcosis in a fox-hound hunt worker, UK	NO CONTROL GROUP
**33**	G. Garippa, et al.	Parassitologia, 46, 4, 387-91	Cystic echinococcosis in Italy from the 1950s to present	NO DATA ON PATIENTS
**34**	R. Neghina, et al.	Vector Borne Zoonotic Dis, 11, 8, 993-9	Cystic echinococcosis in Romania: the pediatric approach	NO CONTROL GROUP
**35**	M. L. de la Rue	Rev Inst Med Trop Sao Paulo, 50, 1, 53-6	Cystic echinococcosis in southern Brazil	NO DATA ON PATIENTS
**36**	F. A. Rojo-Vazquez, et al.	PLoS Negl Trop Dis, 5, 1, e893	Cystic echinococcosis in Spain: current situation and relevance for other endemic areas in Europe	REVIEW
**37**	E. Anna Rita, et al.	Cestode Zoonoses: Echinococcosis and	Cystic echinococcosis in the Mediterranean basin	REVIEW

		Cysticercosis: An Emergent and Global Problem, 341, 41		
**38**	S. Lahmar, et al.	Ann Trop Med Parasitol, 103, 7, 593-604	Cystic echinococcosis in Tunisia: analysis of hydatid cysts that have been surgically removed from patients	NO CONTROL GROUP
**39**	J. C. González Peralta, et al.	Revista de Medicina Veterinaria, 89, 2, 41-44	Cystic echinococcosis on the northeast of the provincial Reserve of Pampa de Achala, Córdoba, Argentina	NO RISK FACTORS DESCRIBED
**40**	A. Hernandez, et al.	Parasitology, 130, Pt 4, 455-60	Cystic echinococcosis: analysis of the serological profile related to the risk factors in individuals without ultrasound liver changes living in an endemic area of Tacuarembo, Uruguay	NO RISK FACTORS DESCRIBED
**41**	F. M. Haridy, et al.	J Egypt Soc Parasitol, 38, 2, 635-44	Cystic hydatidosis: a zoonotic silent health problem	NOT FOUND ARTICLE
**42**	J. B. Koea	N Z Med J, 121, 1277, 61-9	Cystic lesions of the liver: 6 years of surgical management in New Zealand	NO RISK FACTORS DESCRIBED
**43**	M. MOSAYEBI, et al.	Iranian Journal of Parasitology, 8, 4, 510-515	Differential Genomics Output and Susceptibility of Iranian Patients with Unilocular Hydatidose	NO RISK FACTORS DESCRIBED
**44**	A. J. Finkel	Jama, 221, 10, 1172	Dog feces as vector of human disease	REVIEW
**45**	F. Van Kesteren, et al.	Parasitology, 140, 13, 1674-84	Dog ownership, dog behaviour and transmission of Echinococcus spp. in the Alay Valley, southern Kyrgyzstan	NO DATA ON PATIENTS
**46**	P. S. Craig, et al.	Emerging Infectious Diseases, 14, 10, 1674-1675	Echinococcoses and Tibetan Communities	REVIEW
**47**	B. B. Alessandra Siracusano, Federica Delunardo, Paola Margutti, and E. P. e. R. R. Elena Ortona	Not Ist Super Sanità, 17, 2, 3-6	Echinococcosi cistica: un problema sanitario che necessita di approcci multidisciplinari	NOT PRIMARY STUDY
**48**	S. o. P. H. Dept. of Medical Parasitology and Mycology, Tehran University of Medical and I. Sciences	Iranian J Parasitol, 4, 2, 1-16	Echinococcosis /hydatidosis in Iran	REVIEW

**49**	E. Golab and M. P. Czarkowski	Przegl Epidemiol, 68, 2, 279-82, 379-81	Echinococcosis and cysticercosis in Poland in 2012	NO CONTROL GROUP
**50**	P. M. Schantz	Am J Epidemiol, 106, 5, 370-9	Echinococcosis in American Indians living in Arizona and New Mexico: a review of recent studies	NO RISK FACTORS DESCRIBED
**51**	Z. Wang, et al.	Ecohealth, 5, 2, 115-26	Echinococcosis in China, a review of the epidemiology of Echinococcus spp	REVIEW
**52**	Y. R. Yang, et al.	Trans R Soc Trop Med Hyg, 102, 4, 319-28	Echinococcosis in Ningxia Hui Autonomous Region, northwest China	REVIEW
**53**	D. Doicescu, et al.	Journal of Environmental Protection and Ecology, 14, 4, 1836-1844	Echinococcosis in the County of Constanta	NO CONTROL GROUP
**54**	P. R. Torgerson, et al.	Trop Med Int Health, 14, 3, 341-8	Echinococcosis, toxocarosis and toxoplasmosis screening in a rural community in eastern Kazakhstan	NO CONTROL GROUP
**55**	A. Dakkak	Vet Parasitol, 174, 1-2, 2-11	Echinococcosis/hydatidosis: a severe threat in Mediterranean countries	REVIEW
**56**	L. Montinaro, et al.	G Ital Med Lav Ergon, 26, 3, 202-7	Echinococcosis: a persistent endemic	NO RISK FACTORS DESCRIBED
**57**	M. Farahmand and M. Yadollahi	Int J Occup Environ Med, 1, 2, 88-91	Echinococcosis: an occupational disease	NO CONTROL GROUP
**58**	M. Chrieki	Am Fam Physician, 66, 5, 817-20	Echinococcosis--an emerging parasite in the immigrant population	NO RISK FACTORS DESCRIBED
**59**	D. J. Jenkins	Parasitol Int, 55 Suppl, S203-6	Echinococcus granulosus in Australia, widespread and doing well!	REVIEW
**60**	A. Virga and S. Giannetto	Large Animals Review, 4, 1, 71-72	Echinococcus granulosus in sheep dogs in western Sicily.	NO DATA ON PATIENTS
**61**	D. J. Jenkins and B. Morris	Aust Vet J, 81, 1-2, 81-5	Echinococcus granulosus in wildlife in and around the Kosciuszko National Park, south-eastern Australia	NO DATA ON PATIENTS
**62**	Y. R. Yang, et al.	PLoS Negl Trop Dis, 3, 4, e426	Echinococcus granulosus infection and options for control of cystic echinococcosis in Tibetan communities of Western Sichuan Province, China	NO DATA ON PATIENTS

**63**	B. Goga	Praxis Veterinaria (Milano), 31, 4, 7-13	Echinococcus granulosus infection in dogs: should prevention and control of cystic echinococcosis begin here?	NO DATA ON PATIENTS
**64**	G. Acosta-Jamett, et al.	Vet Parasitol, 169, 1-2, 102-10	Echinococcus granulosus infection in humans and livestock in the Coquimbo region, north-central Chile	NO RISK FACTORS DESCRIBED
**65**	A. Bchir, et al.	Lancet, 2, 8560, 684	Echotomographic evidence for a highly endemic focus of hydatidosis in central Tunisia	NO RISK FACTORS DESCRIBED
**66**	B. Chomel	Animals, 4, 3, 434-445	Emerging and Re-Emerging Zoonoses of Dogs and Cats	REVIEW
**67**	P. Dorny, et al.	Vet Parasitol, 163, 3, 196-206	Emerging food-borne parasites	REVIEW
**68**	C. Brown	Rev Sci Tech, 23, 2, 435-42	Emerging zoonoses and pathogens of public health significance--an overview	REVIEW
**69**	R. M. Vorou, et al.	Epidemiol Infect, 135, 8, 1231-47	Emerging zoonoses and vector-borne infections affecting humans in Europe	REVIEW
**70**	C. Fenga and M. Pugliese	G Ital Med Lav Ergon, 35, 4, 347-9	Endemic zoonosis in Mediterranean area	REVIEW
**71**	A. Adinezadeh, et al.	Iranian Journal of Parasitology, 8, 3, 459-466	Endoparasites of Stray Dogs in Mashhad, Khorasan Razavi Province, Northeast Iran with Special Reference to Zoonotic Parasites	NO DATA ON PATIENTS
**72**	J.-A. M. Atkinson, et al.	Global Change Biology, 19, 3, 677-688	Environmental changes impacting Echinococcus transmission: research to support predictive surveillance and control	REVIEW
**73**	J. E. Cheek, et al.	Am J Epidemiol, 174, 11 Suppl, S89-96	Epidemic assistance from the Centers for Disease Control and Prevention involving American Indians and Alaska Natives, 1946-2005	REVIEW
**74**	A. Luo, et al.	J Huazhong Univ Sci Technolog Med Sci, 34, 1, 142-5	Epidemic factors and control of hepatic echinococcosis in Qinghai province	NOT FOUND ARTICLE
**75**	R. G. Cobzaru, et al.	Rev Med Chir Soc Med Nat Iasi, 117, 3, 754-7	Epidemiological aspects of hydatidosis in children, in some areas of north-eastern Romania	NO CONTROL GROUP
**76**	J. J. Chai	Biomed Environ Sci, 8, 2, 122-36	Epidemiological studies on cystic echinococcosis in China--a review	NO RISK FACTORS DESCRIBED

**77**	A. Perez, et al.	Medicina (B Aires), 66, 3, 193-200	Epidemiological surveillance of cystic echinococcosis in dogs, sheep farms and humans in the Rio Negro Province]	NO RISK FACTORS DESCRIBED
**78**	E. Cappello, et al.	World J Gastroenterol, 19, 48, 9351-8	Epidemiology and clinical features of cystic hydatidosis in Western Sicily: a ten-year review	NO CONTROL GROUP
**79**	W. Zhang, et al.	Acta Trop,	Epidemiology and control of echinococcosis in central Asia, with particular reference to the People's Republic of China	REVIEW
**80**	C. N. Macpherson	Vet Parasitol, 54, 1-3, 87-102	Epidemiology and control of parasites in nomadic situations	REVIEW
**81**	C. Palmas, et al.	Boll Ist Sieroter Milan, 68, 1, 82-90	Epidemiology and social/health implications of hydatidosis in Sardinia	NO CONTROL GROUP
**82**	I. Saeed, et al.	J Helminthol, 74, 1, 83-8	Epidemiology of Echinococcus granulosus in Arbil province, northern Iraq, 1990-1998	NO CONTROL GROUP
**83**	V. S. Pandey, et al.	Ann Trop Med Parasitol, 82, 5, 461-70	Epidemiology of hydatidosis/echinococcosis in Ouarzazate, the pre-Saharian region of Morocco	NO DATA ON PATIENTS
**84**	M. Antoniou, et al.	Am J Trop Med Hyg, 66, 1, 80-5	Fourteen-year seroepidemiological study of zoonoses in a Greek village	NO RISK FACTORS DESCRIBED
**85**	A. L. Lamy, et al.	J Pediatr Surg, 28, 9, 1140-3	Giant hydatid lung cysts in the Canadian northwest: outcome of conservative treatment in three children	NO CONTROL GROUP
**86**	M. Inan, et al.	Saudi Med J, 28, 4, 555-8	Hepatic hydatid disease in children and adults living in different areas in Turkey	NO CONTROL GROUP
**87**	R. Orlando, et al.	Recenti Prog Med, 79, 11, 452-4	Hepatic hydatidosis in Campania. Preliminary data on its spread and risk factors	NOT FOUND ARTICLE
**88**	Noya-Alarcon, et al.	Boletin de Malariolog¨ªa y Salud Ambiental,	Hidatidosis poliquistica autoctona en dos pacientes Yanomami en el Alto Orinoco, Amazonas, Venezuela	NO CONTROL GROUP
**89**	B. Gultepe, et al.	Korean J Parasitol, 52, 1, 47-9	Higher seroprevalence of hepatitis B virus antigen in patients with cystic hydatid disease than in patients referred to internal medicine clinics in Turkey	NO RISK FACTORS DESCRIBED
**90**	N. L. Gilbert, et al.	Can J Public Health, 101, 4, 337-40	Hospitalization for trichinellosis and echinococcosis in Canada, 2001-2005: the tip of the iceberg?	NO CONTROL GROUP
**91**	K. Wahlers, et al.	Acta Trop, 120, 3, 179-84	Human cystic echinococcosis in South Africa	NO RISK FACTORS

				DESCRIBED
**92**	H. M. Hajipirloo, et al.	Iran J Parasitol, 8, 2, 323-6	Human cystic echinococcosis in west azerbaijan, northwest iran: a retrospective hospital based survey from 2000 to 2009	NO CONTROL GROUP
**93**	G. Battelli, et al.	Parassitologia, 46, 4, 415-6	Human echinococcosis in the Emilia-Romagna region (northern Italy) in the years 1997 to 2002: an updating	NO RISK FACTORS DESCRIBED
**94**	M. C. ELISSONDO, et al.	Parasitología latinoamericana, 57, 124-128	Human hydatidosis in Mar del Plata, Buenos Aires Province, Argentina, (1992-1995): A preliminary study	NO CONTROL GROUP
**95**	M. Conchedda, et al.	Parassitologia, 27, 3, 225-45	Human hydatidosis in Sardinia. Epidemiologic study of the cases operated on from 1974 to 1981	NOT FOUND ARTICLE
**96**	N. A. Ahmadi and F. Badi	Trop Biomed, 28, 2, 450-6	Human hydatidosis in Tehran, Iran: a retrospective epidemiological study of surgical cases between 1999 and 2009 at two university medical centers	NO CONTROL GROUP
**97**	E. Larrieu, et al.	Rev Inst Med Trop Sao Paulo, 31, 4, 267-70	Human hydatidosis: field echography for the determination of groups at high risk in the evaluation of a control program	NO RISK FACTORS DESCRIBED
**98**	T. D. Sharma and N. L. Chitkara	Indian J Med Res, 51, 1015-8	HYDATID DISEASE IN AMRITSAR: A STUDY OF POTENTIAL HUMAN RISK	NO DATA ON PATIENTS
**99**	B. P. Kalani, et al.	Ann Trop Paediatr, 4, 3, 195-9	Hydatid disease in children in Libya	NOT FOUND ARTICLE
**100**	M. J. Burridge and C. W. Schwabe	Am J Trop Med Hyg, 26, 2, 258-65	Hydatid disease in New Zealand: an epidemiological study of transmission among Maoris	NO DATA ON PATIENTS
**101**	M. J. Burridge, et al.	N Z Med J, 85, 583, 173-7	Hydatid disease in New Zealand: changing patterns in human infection, 1878-1972	NO RISK FACTORS DESCRIBED
**102**	P. Riengchan, et al.	J Med Assoc Thai, 87, 6, 725-9	Hydatid disease of the liver: the first indigenous case in Thailand and review of the literature	REVIEW
**103**	A. G. Barbour, et al.	Am J Trop Med Hyg, 27, 1 Pt 1, 94-100	Hydatid disease screening: Sanpete County, Utah, 1971-1976	NO CONTROL GROUP
**104**	A. G. Ghoshal, et al.	J Assoc Physicians India, 60, 12-6	Hydatid lung disease: an analysis of five years cumulative data from Kolkata	NO CONTROL GROUP
**105**	M. C. Dopchiz, et al.	Rev Soc Bras Med Trop,	Hydatidosis cases in one of Mar del Plata City hospitals,	NO RISK

		40, 6, 635-9	Buenos Aires, Argentina	FACTORS DESCRIBED
**106**	E. Larrieu, et al.	Rev Sanid Hig Publica (Madr), 67, 5, 377-84	Hydatidosis control in the province of Rio Negro, Argentina: development of primary care programs	NO RISK FACTORS DESCRIBED
**107**	E. Larrieu, et al.	Rev Sanid Hig Publica (Madr), 68, 1, 197-202	Hydatidosis control in the province of Rio Negro, Argentina: evaluation of the veterinary health care activities.	NO RISK FACTORS DESCRIBED
**108**	T. C. Beard	Australian Veterinary Journal, 55, 3, 131-135	HYDATIDS IN AUSTRALIA — THE PRESENT POSITION IN MAN	NO RISK FACTORS DESCRIBED
**109**	J. M. Pinon and G. Dropsy	Biomedicine, 25, 9, 341-4	Immunological study of hydatidosis. I. Evaluation of the test of immuno-electrodiffusion in the humoral study of human hydatidosis	NO RISK FACTORS DESCRIBED
**110**	Y. R. Yang, et al.	Parasit Vectors, 5, 146	Impact of anthropogenic and natural environmental changes on Echinococcus transmission in Ningxia Hui Autonomous Region, the People's Republic of China	REVIEW
**111**	S. M. Donovan, et al.	Am J Trop Med Hyg, 53, 6, 668-71	Imported echinococcosis in southern California	REVIEW
**112**	A. I. Molineri, et al.	Rev Argent Microbiol, 46, 1, 7-13	Knowledge of zoonoses transmission routes and of the species concerned among rural workers	NO RISK FACTORS DESCRIBED
**113**	Dawit Gebremichael, et al.	Global Veterinaria, 11, 3, 272-279	Knowledge, Attitude and Practices of Hydatidosis in Pastoral Community with Relation to Public Health Risks in Ayssaita, Northeastern of Ethiopia	NO RISK FACTORS DESCRIBED
**114**	R. Mzali, et al.	Tunis Med, 85, 5, 367-70	Liver cystic echinococcosis: which cysts are correlated with false negative indirect passive hemagglutination (IHA)?	NO RISK FACTORS DESCRIBED
**115**	A. E. Utuk, et al.	Acta Trop, 107, 2, 192-4	Molecular genetic characterization of different isolates of Echinococcus granulosus in east and southeast regions of Turkey	NO RISK FACTORS DESCRIBED
**116**	C. N. Macpherson, et al.	Ann Trop Med Parasitol, 83, 5, 489-97	Observations on human echinococcosis (hydatidosis) and evaluation of transmission factors in the Maasai of northern	NO RISK FACTORS

			Tanzania	DESCRIBED
**117**	J. Heptonstall, et al.	Hunter's diseases of occupations, Ed. 9, 489-520	Occupation and infectious diseases	NOT FOUND ARTICLE
**118**	O. Jones, et al.	Ethiop. J. Health Dev, 26, 1, 43-48	Occurrence of bovine hydatidosis and evaluation of its risk to humans in traditional communities of Southern Region of Ethiopia	NO DATA ON PATIENTS
**119**	E. J. Jenkins, et al.	Vet Parasitol, 182, 1, 54-69	Old problems on a new playing field: Helminth zoonoses transmitted among dogs, wildlife, and people in a changing northern climate	REVIEW
**120**	S.-K. Hugues, et al.	Epidemiology, 23, 5S,	P-123: Seroprevalence of Zoonotic Infections in Two Native Communities	NO RISK FACTORS DESCRIBED
**121**	R. C. Thompson	Int J Parasitol, 43, 12-13, 1079-88	Parasite zoonoses and wildlife: One Health, spillover and human activity	REVIEW
**122**	D. Sturchler, et al.	Ann Trop Med Parasitol, 81, 3, 291-9	Parasitic infections in Yakima Indians	NO RISK FACTORS DESCRIBED
**123**	A. J. Hughes and B. A. Biggs	Intern Med J, 32, 11, 541-53	Parasitic worms of the central nervous system: an Australian perspective	REVIEW
**124**	J. M. Schurer, et al.	PLoS Neglected Tropical Diseases, 7, 3, e2141	Parasitic Zoonoses: One Health Surveillance in Northern Saskatchewan	NO RISK FACTORS DESCRIBED
**125**	N. Akritidis	Clin Microbiol Infect, 17, 3, 331-5	Parasitic, fungal and prion zoonoses: an expanding universe of candidates for human disease	REVIEW
**126**	S. J. Condie, et al.	Public Health, 95, 1, 28-35	Participation in a community program to prevent hydatid disease	NO RISK FACTORS DESCRIBED
**127**	J. M. Schurer, et al.	Am J Trop Med Hyg, 90, 6, 1184-90	People, pets, and parasites: one health surveillance in southeastern Saskatchewan	NO RISK FACTORS DESCRIBED
**128**	C. N. Macpherson and R. Milner	Acta Trop, 85, 2, 203-9	Performance characteristics and quality control of community based ultrasound surveys for cystic and alveolar echinococcosis	REVIEW

**129**	G. Juckett	Am Fam Physician, 56, 7, 1763-74, 1777-8	Pets and Parasites	REVIEW
**130**	H. Benchaoui	Handb Exp Pharmacol, 199, 113-38	Population medicine and control of epidemics	REVIEW
**131**	J. R. Crellin, et al.	Am J Epidemiol, 116, 3, 463-74	Possible factors influencing distribution and prevalence of Echinococcus granulosus in Utah	NO DATA ON PATIENTS
**132**	P. L. Moro, et al.	Rev Gastroenterol Peru, 28, 1, 43-9	Practices, knowledge and attitudes about human hydatidosis in Peru	NO DATA ON PATIENTS
**133**	H. O. El Malki, et al.	BMC Surg, 10, 16	Predictive model of biliocystic communication in liver hydatid cysts using classification and regression tree analysis	NO RISK FACTORS DESCRIBED
**134**	Octavius Jones, et al.	Journal of Public Health and Epidemiology, 3, 11, 550-555	Prevalence of Dog Gastrointestinal Parasites and Risk Perception of Zoonotic Infection by Dog Owners in Wondo Genet, Southern Ethiopia	NO DATA ON PATIENTS
**135**	S. M. Soria, et al.	Acta Gastroenterol Latinoam, 36, 4, 174-81	Prevalence of liver diseases in a small rural community isolated in the mountain heights: clinical, biochemical and ultrasonographic study	NO RISK FACTORS DESCRIBED
**136**	S. Jimenez, et al.	Acta Trop, 83, 3, 213-21	Progress in control of cystic echinococcosis in La Rioja, Spain: decline in infection prevalences in human and animal hosts and economic costs and benefits	NO RISK FACTORS DESCRIBED
**137**	M. Kachani, et al.	Acta Trop, 85, 2, 263-9	Public health education/importance and experience from the field. Educational impact of community-based ultrasound screening surveys	NO RISK FACTORS DESCRIBED
**138**	S. H. Fatimi, et al.	ANZ J Surg, 77, 9, 749-51	Pulmonary hydatidosis: clinical profile and follow up from an endemic region	NO RISK FACTORS DESCRIBED
**139**	I. Serra, et al.	Bol Chil Parasitol, 51, 1-2, 3-12	Regional analysis of human and animal hydatidosis in Chile, 1989-1993	NO RISK FACTORS DESCRIBED
**140**	S. Gulsun, et al.	Iran J Parasitol, 5, 3, 20-6	Retrospective analysis of echinococcosis in an endemic region of Turkey, a review of 193 cases	NO RISK FACTORS DESCRIBED
**141**	D. Brundu, et al.	Acta Trop, 140, 91-6	Retrospective study of human cystic echinococcosis in Italy	NO RISK

			based on the analysis of hospital discharge records between 2001 and 2012	FACTORS DESCRIBED
**142**	A. T. AU Amfim, I. P.; Simion, V. E.; Parvu, M.	Bulletin UASVM, Veterinary Medicine, 67, 2,	Retrospective Survey of Human Hydatidosis in Bucharest Based on Hospital and Family Doctors Records	NO RISK FACTORS DESCRIBED
**143**	A. I. Youssef and S. Uga	Trop Med Health, 42, 1, 3-14	Review of parasitic zoonoses in egypt	REVIEW
**144**	Q. Wang, et al.	Infect Dis Poverty, 3, 1, 3	Review of risk factors for human echinococcosis prevalence on the Qinghai-Tibet Plateau, China: a prospective for control options	REVIEW
**145**	P. L. Moro, et al.	Vet Parasitol, 130, 1-2, 99-104	Risk factors for canine echinococcosis in an endemic area of Peru	NO DATA ON PATIENTS
**146**	S. Ernst and J. Robin	Bol Chil Parasitol, 35, 3-4, 75-6	Risk level conditioning factors of hydatid disease in the Province of Valdivia, Chile (author's transl)	REVIEW
**147**	B. I. Pavlin, et al.	Emerg Infect Dis, 15, 11, 1721-6	Risk of importing zoonotic diseases through wildlife trade, United States	NOT PRIMARY STUDY
**148**	E. Gkogka, et al.	Emerging Infectious Diseases, 17, 9, 1581-1590	Risk-based Estimate of Effect of Foodborne Diseases on Public Health, Greece	NO RISK FACTORS DESCRIBED
**149**	D. N. Durrheim, et al.	Journal of Travel Medicine, 8, 4, 176-191	Safety of Travel in South Africa: The Kruger National Park	NO RISK FACTORS DESCRIBED
**150**	Erick V. G. Komba, et al.	Tanzania Journal of Health Research, 14, 2,	Sanitary practices and occurrence of zoonotic conditions in cattle at slaughter in Morogoro Municipality, Tanzania: implications for public health	NO DATA ON PATIENTS
**151**	...	EFSA Journal, 11, 1,	Scientific Opinion on Review of the European Union Summary Report on trends and sources of zoonoses, zoonotic agents and food-borne outbreaks—Terms of reference 2 to 7	NOT PRIMARY STUDY
**152**	R. Baldelli, et al.	Ann Ig, 7, 6, 445-50	Seroepidemiological studies on zoonoses in farm workers in Apulia	NO RISK FACTORS DESCRIBED
**153**	Z. Heidari, et al.	Iranian Journal of Parasitology, 6, 3, 19-25	Seroepidemiological Study of Human Hydatidosis in Meshkinshahr District, Ardabil Province, Iran	NO RISK FACTORS

				DESCRIBED
**154**	A. A. Yacoub, et al.	East Mediterr Health J, 12, 1-2, 112-8	Seroepidemiology of selected zoonotic infections in Basra region of Iraq	NO RISK FACTORS DESCRIBED
**155**	N. Mlika, et al.	Am J Trop Med Hyg, 33, 6, 1182-4	Serologic survey of human hydatid disease in high risk populations from central Tunisia. Preliminary results	NO RISK FACTORS DESCRIBED
**156**	Y. R. Yang, et al.	Trop Med Int Health, 13, 8, 1086-94	Serological prevalence of echinococcosis and risk factors for infection among children in rural communities of southern Ningxia, China	NO RISK FACTORS DESCRIBED
**157**	A. Bchir, et al.	Ann Trop Med Parasitol, 82, 3, 271-3	Serological screening for hydatidosis in households of surgical cases in central Tunisia	NO RISK FACTORS DESCRIBED
**158**	A. Rakhshanpour, et al.	Iran J Parasitol, 7, 3, 10-5	Seroprevalence of human hydatidosis using ELISA method in qom province, central iran	NO RISK FACTORS DESCRIBED
**159**	B. Levesque, et al.	Diagn Microbiol Infect Dis, 59, 3, 283-6	Seroprevalence of zoonoses in a Cree community (Canada)	NO RISK FACTORS DESCRIBED
**160**	F. M. Haridy, et al.	J Egypt Soc Parasitol, 30, 2, 423-9	Sheep-dog-man. The risk zoonotic cycle in hydatidosis	NOT FOUND ARTICLE
**161**	A. Sanli, et al.	Afr Health Sci, 11 Suppl 1, S82-5	Social factors associated with pulmonary hydatid cyst in Aegean, Turkey	NO RISK FACTORS DESCRIBED
**162**	V. Lungu	Revista Scientia Parasitologica, 10, 1/2, 21-25	Some aspects of hydatidosis humanis in Republic of Moldova.	NO RISK FACTORS DESCRIBED
**163**	F. C. Baldock, et al.	Trans R Soc Trop Med Hyg, 79, 2, 238-41	Strain identification of Echinococcus granulosus in determining origin of infection in a case of human hydatid disease in Australia	NO RISK FACTORS DESCRIBED
**164**	A. Seimenis and D. Tabbaa	Vet Ital, 50, 2, 131-6	Stray animal populations and public health in the South Mediterranean and the Middle East regions	NO RISK FACTORS DESCRIBED

**165**	K. Tasdemir, et al.	J Card Surg, 24, 3, 281-4	Surgical approach to the management of cardiovascular echinococcosis	NO RISK FACTORS DESCRIBED
**166**	M. Dakak, et al.	J R Coll Surg Edinb, 47, 5, 689-92	Surgical treatment for pulmonary hydatidosis (a review of 422 cases)	REVIEW
**167**	A. I. Begin, S.; Vandenbroucke-Menu, F.; and R. P. Letourneau, M.; Roy, A.; Dagenais, M.; Lapointe, R.	Hepato-Pancreato-Biliary Association, 12, 1, 147-148	Surgical treatment of liver hydatid cysts: A challenging experience from a non-endemic country	NO RISK FACTORS DESCRIBED
**168**	J. A. Atkinson, et al.	PLoS Negl Trop Dis, 7, 8, e2386	Synthesising 30 years of mathematical modelling of Echinococcus transmission	REVIEW
**169**	B. S. Shaikenov, et al.	Acta Trop, 85, 2, 287-93	The changing epidemiology of echinococcosis in Kazakhstan due to transformation of farming practices	NO DATA ON PATIENTS
**170**	R. J. Yaghan, et al.	Saudi Med J, 25, 7, 886-9	The clinical and epidemiological features of hydatid disease in Northern Jordan	NO DATA ON PATIENTS
**171**	D. O. Ehizibolo, et al.	African Journal of Biomedical Research, 14, 2, 81-88	The Control of Neglected Zoonotic Diseases in Nigeria through Animal Intervention	REVIEW
**172**	T. C. Beard	Bull World Health Organ, 48, 6, 653-60	The elimination of echinococcosis from Iceland	NOT PRIMARY STUDY
**173**	M. F. Stallbaumer, et al.	J Hyg (Lond), 96, 1, 121-7	The epidemiology of hydatid disease in England and Wales	NO CONTROL GROUP
**174**	M. Matsuhashi, et al.	Transfusion, 50, 5, 1126-30	The first case of alloantibody against human platelet antigen-15b in Japan: possible alloimmunization by a hydatidiform mole	NO RISK FACTORS DESCRIBED
**175**	I. D. Robertson, et al.	Int J Parasitol, 30, 12-13, 1369-77	The role of companion animals in the emergence of parasitic zoonoses	REVIEW
**176**	D. J. Jenkins and N. A. Craig	Med J Aust, 157, 11-12, 754-6	The role of foxes Vulpes vulpes in the epidemiology of Echinococcus granulosus in urban environments	NO RISK FACTORS DESCRIBED
**177**	John M. Kagira and P. W. N. Kanyari	SCIENTIA PARASITOLOGICA, 11,	The role of veterinary and medical personnel in the control of zoonoses in urban settlements on the shores of Lake Victoria,	NO RISK FACTORS

		2, 61-66	Kenya	DESCRIBED
**178**	A. M. Qaqish, et al.	Ann Trop Med Parasitol, 97, 5, 511-20	The seroprevalences of cystic echinococcosis, and the associated risk factors, in rural-agricultural, bedouin and semi-bedouin communities in Jordan	NOT FOUND ARTICLE
**179**	I. F. De Villiers	S Afr Med J, 31, 28, 700-2	The threat of hydatid disease to the South African citizen	NO CONTROL GROUP
**180**	D. G. Heppner, et al.	New England Journal of Medicine, 328, 14, 1061-1068	The Threat of Infectious Diseases in Somalia	NO RISK FACTORS DESCRIBED
**181**	A. Ito, et al.	Southeast Asian J Trop Med Public Health, 37 Suppl 3, 82-90	Towards the international collaboration for detection, surveillance and control of taeniasis/ cysticercosis and echinococcosis in Asia and the Pacific	REVIEW
**182**	E. J. Jenkins, et al.	Adv Parasitol, 82, 33-204	Tradition and transition: parasitic zoonoses of people and animals in Alaska, northern Canada, and Greenland	REVIEW
**183**	P. Martín-Dávila, et al.	Clinical Microbiology Reviews, 21, 1, 60-96	Transmission of Tropical and Geographically Restricted Infections during Solid-Organ Transplantation	NO RISK FACTORS DESCRIBED
**184**	K. Gunay, et al.	J Trauma, 46, 1, 164-7	Traumatic rupture of hydatid cysts: a 12-year experience from an endemic region	NO RISK FACTORS DESCRIBED
**185**	L. J. Castrodale, et al.	Am J Trop Med Hyg, 66, 3, 325-7	Two atypical cases of cystic echinococcosis (Echinococcus granulosus) in Alaska, 1999	NO CONTROL GROUP
**186**	Y. R. Yang, et al.	Am J Trop Med Hyg, 74, 3, 487-94	Unique family clustering of human echinococcosis cases in a chinese community	NO RISK FACTORS DESCRIBED
**187**	J. Eckert	Schweiz Med Wochenschr, 127, 39, 1598-608	Veterinary parasitology and human health	REVIEW
**188**	S. Nithiuthai, et al.	Vet Parasitol, 126, 1-2, 167-93	Waterborne zoonotic helminthiases	REVIEW
**189**	J. E. Pearson	Vet Ital, 42, 4, 293	Worldwide risks of animal diseases: introduction	REVIEW
**190**	A. M. Seimenis	Vet Ital, 44, 4, 573-6	Zoonotic diseases in the Mediterranean region: a brief introduction	REVIEW

**191**	K. Hueffer, et al.	Int J Circumpolar Health, 72,	Zoonotic infections in Alaska: disease prevalence, potential impact of climate change and recommended actions for earlier disease detection, research, prevention and control	REVIEW
**192**	H. Sampasa-Kanyinga, et al.	Can J Infect Dis Med Microbiol, 24, 2, 79-84	Zoonotic infections in communities of the James Bay Cree territory: An overview of seroprevalence	NO RISK FACTORS DESCRIBED
**193**	H. Sampasa-Kanyinga, et al.	Vector Borne Zoonotic Dis, 12, 6, 473-81	Zoonotic infections in native communities of James Bay, Canada	NO RISK FACTORS DESCRIBED
**194**	D.P Jordanova, et al.	Eur J Clin Microbiol Infect Dis, 34:1423–1428	Cystic echinococcosis in Bulgaria 1996-2013, with emphasis on childhood infections.	NO CONTROL GROUP
**195**	M.H Kohansal, et al.	Iran J Public Health, Vol. 44, No.9, Sep 2015, pp.1277-1282	Human Cystic Echinococcosis in Zanjan Area, Northwest Iran: A Retrospective Hospital Based Survey between 2007 and 2013.	NO CONTROL GROUP
**196**	M. Ahmadinejad, et al.	International journal of Advanced Biological and Biomedical Research, 3(4): 320–325	Human Cystic Echinococcosis in Lorestan province, Southwest Iran: a retrospective epidemiological study of surgical cases during a 10 years period (2005-2014).	NO CONTROL GROUP
**197**	Z. Andalib Aliabadi, et al.	Iran J Parasitol: Vol. 10, No. 4, pp.658-662	Human hydatidosis /echinococosis in north eastern Iran from 2003-2012.	NO CONTROL GROUP
**198**	D. Brundu, et al.	Acta Tropica, 140: 91–96	Retrospective study of human cystic echinococcosis in Italy based on the analysis of hospital discharge records between 2001 and 2012.	NO CONTROL GROUP
**199**	M. Fazal Karim, et al.	J Infect Dev Ctries, 9(1):070-075	Abdominal cystic echinococcosis in Bangladesh: a hospital-based study.	NO CONTROL GROUP
**200**	A.M.J McFadden, et al.	Zoonoses and Public Health, 63, 138–151	Use of Multicriteria Risk Ranking of Zoonotic Diseases in a Developing Country: Case Study of Mongolia	NO CONTROL GROUP
**201**	A.M Cadavid Restrepo, et al.	Infectious Diseases of Poverty, 5:13	The landscape epidemiology of echinococcoses	REVIEW
**202**	M. Fallah and K. Rahmati	Tropical Medicine and International Health. 9th European Congress on Tropical Medicine and	A retrospective study on 182 cases human hydatidosis based on hospital records, from 2006 to 2013 in Hamadan, West of Iran.	NO CONTROL GROUP

		International Health. Basel, Switzerland.		
**203**	U. Bakal, et al.	Korean J Parasitol, Vol. 53, No. 6: 785-788	Surgical and Molecular Evaluation of Pediatric Hydatid Cyst Cases in Eastern Turkey	NO CONTROL GROUP
**204**	M.A Asencio, et al.	Jpn. J. Infect. Dis., 68, 106–112, 2015	Seroprevalence Survey of Zoonoses in Extremadura, Southwestern Spain, 2002–2003	NO CONTROL GROUP
**205**	P. Mayor, et al.	Emerging Infectious Diseases, 21 (3)	Polycystic Echinococcosis in Pacas, Amazon Region, Peru	NO DATA ON PATIENTS
**206**	J.M Schurer, et al.	PLOS Neglected Tropical Diseases | DOI:10.1371/journal.pntd.0003883	Echinococcosis: An Economic Evaluation of a Veterinary Public Health Intervention in Rural Canada	NO DATA ON PATIENTS
**207**	I. El Berbri, et al.	Infectious Diseases of Poverty, 4:48	Knowledge, attitudes and practices with regard to the presence, transmission, impact, and control of cystic echinococcosis in Sidin Kacem Province, Morocco.	NO DATA ON PATIENTS
**208**	D. Li, et al.	Acta Tropica, 147: 17–22	Knowledge, attitude, and practices (KAP) and risk factors analysisrelated to cystic echinococcosis among residents in Tibetancommunities, Xiahe County, Gansu Province, China	NO DATA ON PATIENTS
**209**	M. Elamin Ahmed, et al.	ASTMH 63rd anual meeting.	KAP (knowledge, attitude and practice) on echinococcosis first report from central Sudan	NO DATA ON PATIENTS
**210**	R. Chaâbane-Banaoues, et al.	Korean J Parasitol Vol. 54, No. 1: 113-118	Environmental Contamination by Echinococcus granulosus sensu lato Eggs in Relation to Slaughterhouses in Urban and Rural Areas in Tunisia	NO RISK FACTORS DESCRIBED
**211**	A. Jurke, et al.	International Journal of Medical Microbiology, 305 652–662	Serological survey of Bartonella spp., Borrelia burgdorferi, Brucella spp.,Coxiella burnetii, Francisella tularensis, Leptospira spp., Echinococcus,Hanta-, TBE- and XMR-virus infection in employees of two forestryenterprises in North Rhine–Westphalia, Germany, 2011–2013	NO RISK FACTORS DESCRIBED
**212**	W. Zhang, et al.	Acta Tropica, 141:235–243	Epidemiology and control of echinococcosis in central Asia, with particular reference to the People's Republic of China	NOT PRIMARY STUDY
