# Supplementary material for: Potential Risk Factors Associated with Human Cystic Echinococcosis: Systematic Review and Meta-analysis
Source: PLoS Negl Trop Dis. 2016 Nov 7;10(11):e0005114. doi: 10.1371/journal.pntd.0005114 (PMC5098738; doi:10.1371/journal.pntd.0005114)

# CASE-CONTROL studies reporting forest and funnel plot analysis on single potential risk factors.

## Dog ownership

| Study or Subgroup | case Events | case Total | Control Events | Control Total | Weight | Odds Ratio M-H, Random, 95% CI |
|---|---|---|---|---|---|---|
| Abou-Daoud 1964, 90.1 | 23 | 26 | 11 | 44 | 17.8% | 23.00 [5.77, 91.71] |
| Campos-Bueno 2000, 179.3 | 119 | 224 | 8 | 30 | 22.2% | 3.12 [1.33, 7.30] |
| Dowling 2000, 177.1 | 7 | 32 | 37 | 144 | 21.7% | 0.81 [0.32, 2.03] |
| Larrieu 2002, 9.1 | 21 | 71 | 3 | 19 | 18.2% | 2.24 [0.59, 8.51] |
| Moro 2008, 128.4 | 12 | 18 | 20 | 78 | 20.1% | 5.80 [1.92, 17.49] |
| **Total (95% CI)** | | **371** | | **315** | **100.0%** | **3.54 [1.27, 9.85]** |
| Total events | 182 | | 79 | | | |

Heterogeneity: $Tau^2 = 1.04$; $Chi^2 = 17.73$, df = 4 (P = 0.001); $I^2 = 77\%$
Test for overall effect: Z = 2.42 (P = 0.02)

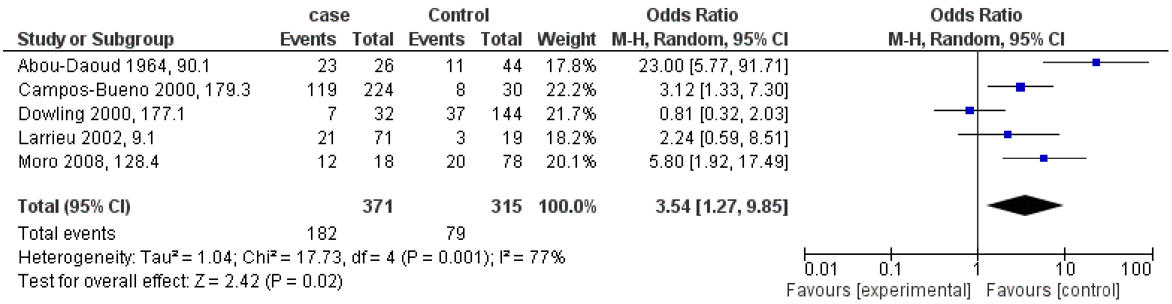


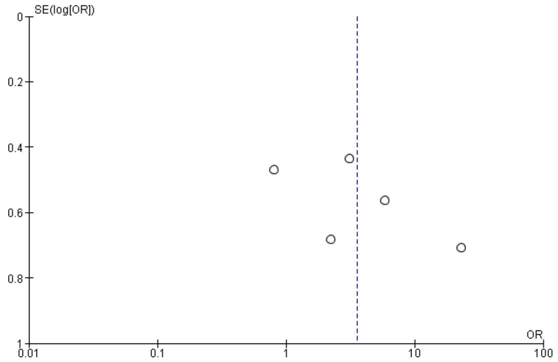


## Feeding dogs with viscera

| Study or Subgroup | case Events | case Total | Control Events | Control Total | Weight | Odds Ratio M-H, Fixed, 95% CI |
|---|---|---|---|---|---|---|
| Campos-Bueno 2000, 179.1 | 111 | 177 | 16 | 77 | 45.8% | 6.41 [3.42, 12.03] |
| Dowling 2000, 177.2 | 0 | 4 | 44 | 172 | 12.4% | 0.32 [0.02, 6.08] |
| Larrieu 2002, 9.4 | 15 | 36 | 9 | 54 | 23.2% | 3.57 [1.35, 9.47] |
| Moro 2008, 128.1 | 14 | 23 | 18 | 73 | 18.6% | 4.75 [1.76, 12.82] |
| **Total (95% CI)** | | **240** | | **376** | **100.0%** | **4.69 [3.02, 7.29]** |
| Total events | 140 | | 87 | | | |

Heterogeneity: $Chi^2 = 4.44$, df = 3 (P = 0.22); $I^2 = 32\%$
Test for overall effect: Z = 6.86 (P < 0.00001)

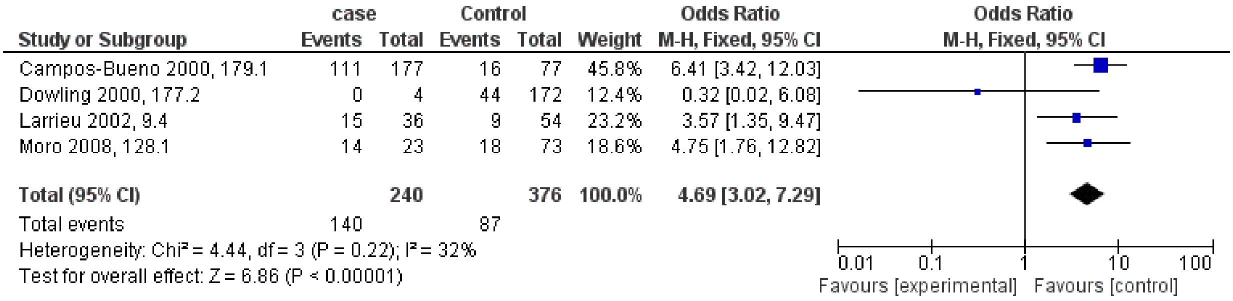


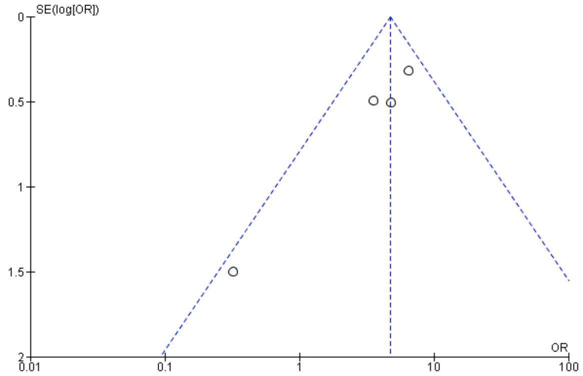

# Dog contact

| Study or Subgroup | case Events | case Total | Control Events | Control Total | Weight | Odds Ratio M-H, Random, 95% CI |
|---|---|---|---|---|---|---|
| Campos-Bueno 2000, 179.2 | 91 | 170 | 36 | 84 | 59.8% | 1.54 [0.91, 2.60] |
| Moro 2008, 128.2 | 31 | 75 | 1 | 21 | 40.2% | 14.09 [1.80, 110.59] |
| **Total (95% CI)** | | **245** | | **105** | **100.0%** | **3.74 [0.41, 33.96]** |
| Total events | 122 | | 37 | | | |

Heterogeneity: Tau² = 2.04; Chi² = 4.47, df = 1 (P = 0.03); I² = 78%
Test for overall effect: Z = 1.17 (P = 0.24)

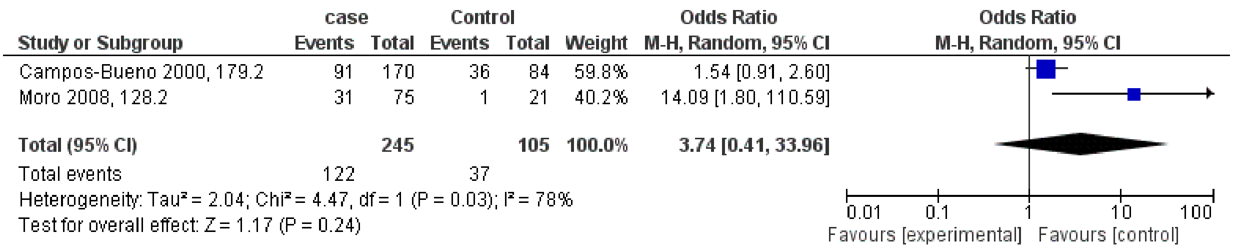


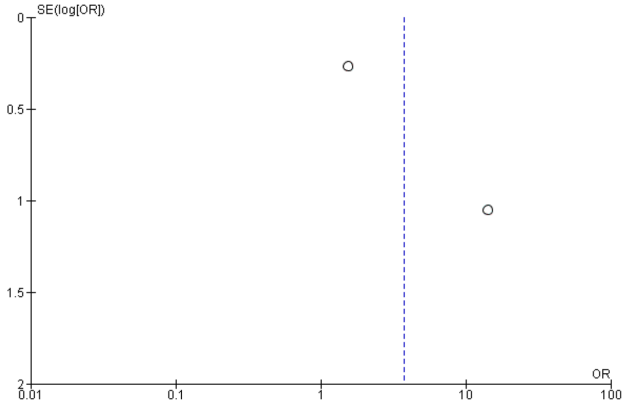


# Dogs free to roam

| Study or Subgroup | case Events | case Total | Control Events | Control Total | Weight | Odds Ratio M-H, Fixed, 95% CI |
|---|---|---|---|---|---|---|
| Larrieu 2002, 9.3 | 17 | 55 | 4 | 32 | 53.8% | 3.13 [0.95, 10.33] |
| Moro 2008, 128.3 | 24 | 42 | 8 | 54 | 46.2% | 7.67 [2.91, 20.19] |
| **Total (95% CI)** | | **97** | | **86** | **100.0%** | **5.23 [2.45, 11.14]** |
| Total events | 41 | | 12 | | | |

Heterogeneity: Chi² = 1.31, df = 1 (P = 0.25); I² = 24%
Test for overall effect: Z = 4.28 (P < 0.0001)

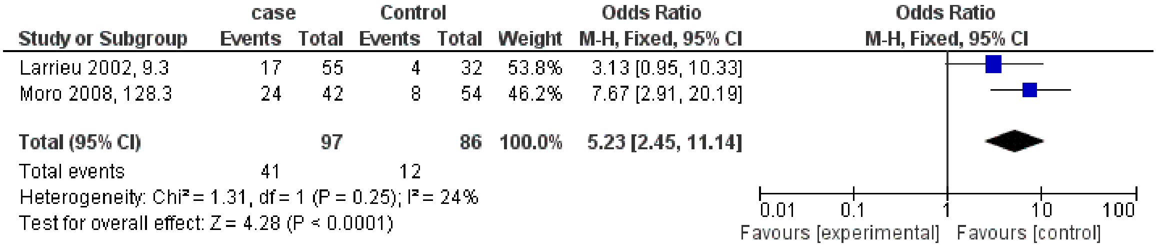


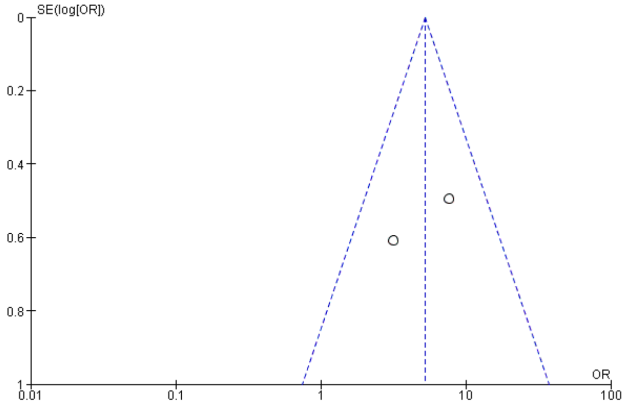

# Dog deworming never or infrequently

| Study or Subgroup | case Events | case Total | Control Events | Control Total | Weight | Odds Ratio M-H, Fixed, 95% CI |
|---|---|---|---|---|---|---|
| Dowling 2000, 177.3 | 41 | 169 | 3 | 7 | 41.1% | 0.43 [0.09, 1.99] |
| Larrieu 2002, 9.2 | 13 | 47 | 8 | 40 | 58.9% | 1.53 [0.56, 4.18] |
| **Total (95% CI)** | | **216** | | **47** | **100.0%** | **1.08 [0.47, 2.49]** |
| Total events | 54 | | 11 | | | |

Heterogeneity: Chi² = 1.86, df = 1 (P = 0.17); I² = 46%
Test for overall effect: Z = 0.17 (P = 0.86)

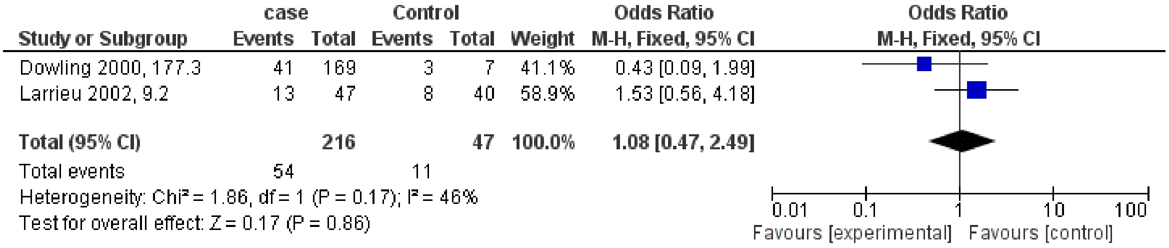


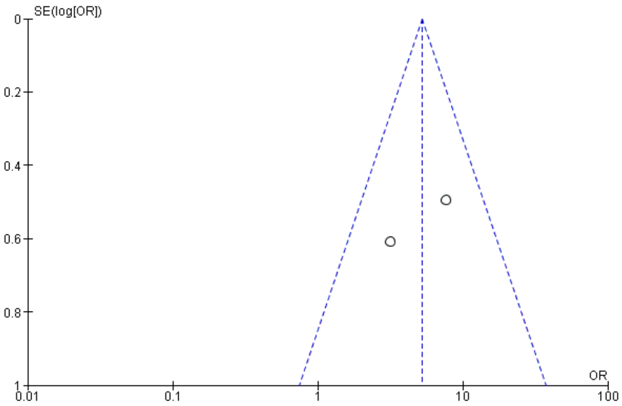


# Slaughter at home

| Study or Subgroup | case Events | case Total | Control Events | Control Total | Weight | Odds Ratio M-H, Fixed, 95% CI |
|---|---|---|---|---|---|---|
| Larrieu 2002, 9.1 | 20 | 60 | 4 | 30 | 63.1% | 3.25 [1.00, 10.59] |
| Moro 2008, 128.1 | 12 | 17 | 20 | 79 | 36.9% | 7.08 [2.22, 22.58] |
| **Total (95% CI)** | | **77** | | **109** | **100.0%** | **4.67 [2.02, 10.78]** |
| Total events | 32 | | 24 | | | |

Heterogeneity: Chi² = 0.86, df = 1 (P = 0.35); I² = 0%
Test for overall effect: Z = 3.60 (P = 0.0003)

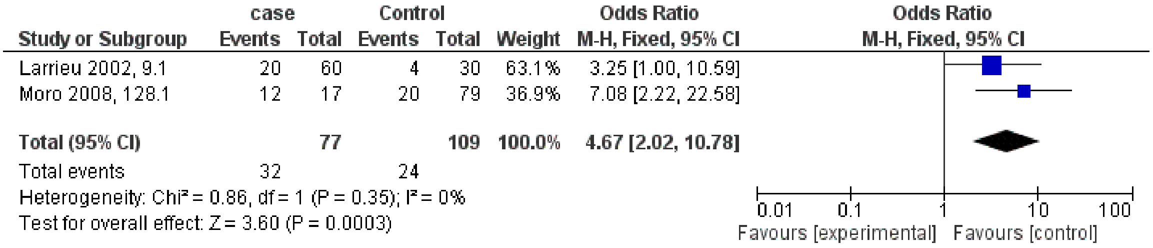


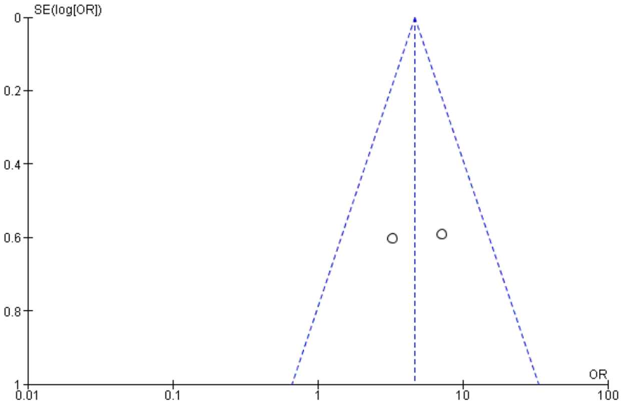

## Slaughterhouse

| Study or Subgroup | case Events | case Total | Control Events | Control Total | Weight | Odds Ratio M-H, Fixed, 95% CI |
|---|---|---|---|---|---|---|
| Larrieu 2002, 9.2 | 13 | 37 | 11 | 53 | 91.0% | 2.07 [0.80, 5.33] |
| Moro 2008, 128.2 | 4 | 5 | 28 | 91 | 9.0% | 9.00 [0.96, 84.21] |
| **Total (95% CI)** | | **42** | | **144** | **100.0%** | **2.70 [1.15, 6.30]** |
| Total events | 17 | | 39 | | | |

Heterogeneity: Chi² = 1.42, df = 1 (P = 0.23); I² = 29%
Test for overall effect: Z = 2.29 (P = 0.02)

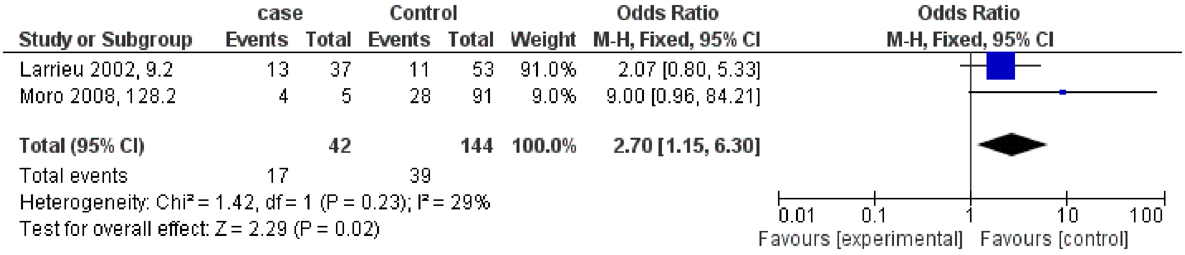


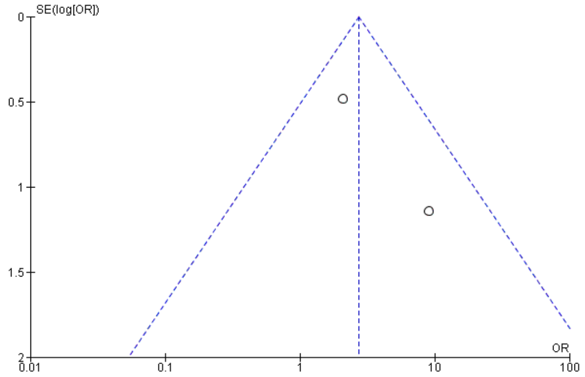


## Living in rural areas

| Study or Subgroup | case Events | case Total | Control Events | Control Total | Weight | Odds Ratio M-H, Fixed, 95% CI |
|---|---|---|---|---|---|---|
| Campos-Bueno 2000, 179.1 | 86 | 159 | 41 | 95 | 86.2% | 1.55 [0.93, 2.59] |
| Larrieu 2002, 9.1 | 19 | 53 | 5 | 37 | 13.8% | 3.58 [1.19, 10.71] |
| **Total (95% CI)** | | **212** | | **132** | **100.0%** | **1.83 [1.16, 2.90]** |
| Total events | 105 | | 46 | | | |

Heterogeneity: Chi² = 1.83, df = 1 (P = 0.18); I² = 45%
Test for overall effect: Z = 2.59 (P = 0.010)

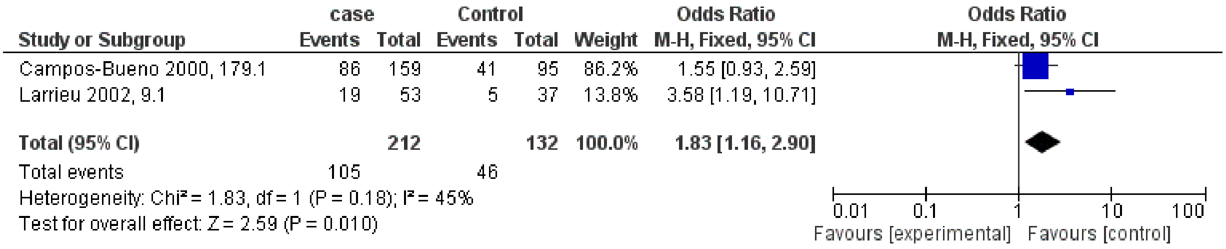


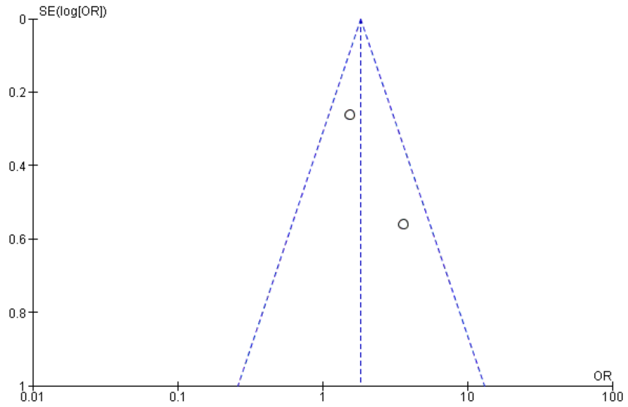

# Herding

| | case | | Control | | | Odds Ratio |
|---|---|---|---|---|---|---|
| Study or Subgroup | Events | Total | Events | Total | Weight | M-H, Random, 95% CI |
| Campos-Bueno 2000, 179.1 | 87 | 163 | 40 | 91 | 17.4% | 1.46 [0.87, 2.44] |
| Dowling 2000, 177.1 | 9 | 43 | 35 | 133 | 13.6% | 0.74 [0.32, 1.70] |
| Larrieu 2002, 9.1 | 14 | 33 | 10 | 57 | 12.0% | 3.46 [1.31, 9.14] |
| Moro 2008, 128.1 | 11 | 37 | 21 | 59 | 12.9% | 0.77 [0.32, 1.85] |
| Moro 2008, 128.3 | 7 | 32 | 11 | 64 | 11.0% | 1.35 [0.47, 3.89] |
| Moro 2008, 128.4 | 2 | 19 | 30 | 77 | 7.2% | 0.18 [0.04, 0.86] |
| Moro 2008, 128.5 | 20 | 41 | 12 | 55 | 12.9% | 3.41 [1.41, 8.27] |
| Moro 2008, 128.6 | 21 | 54 | 11 | 42 | 13.0% | 1.79 [0.74, 4.32] |
| **Total (95% CI)** | | **422** | | **578** | **100.0%** | **1.33 [0.80, 2.21]** |
| Total events | 171 | | 170 | | | |

Heterogeneity: Tau² = 0.32; Chi² = 18.42, df = 7 (P = 0.01); I² = 62%
Test for overall effect: Z = 1.10 (P = 0.27)

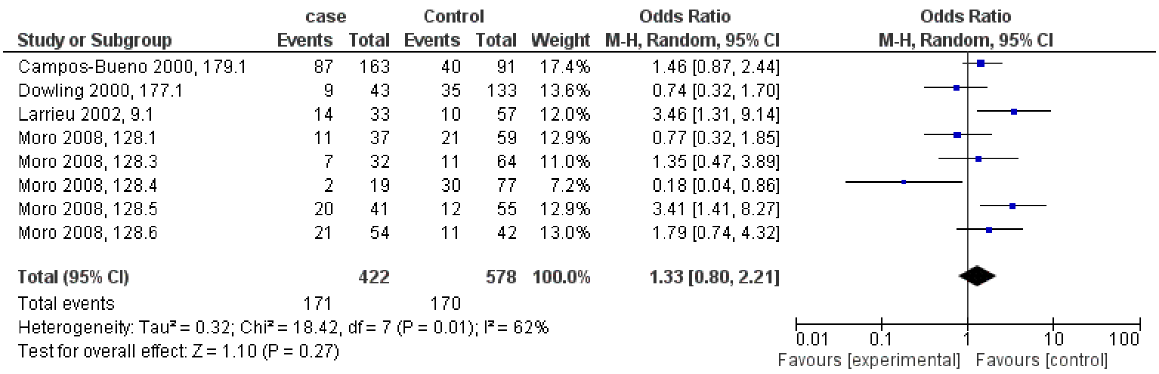


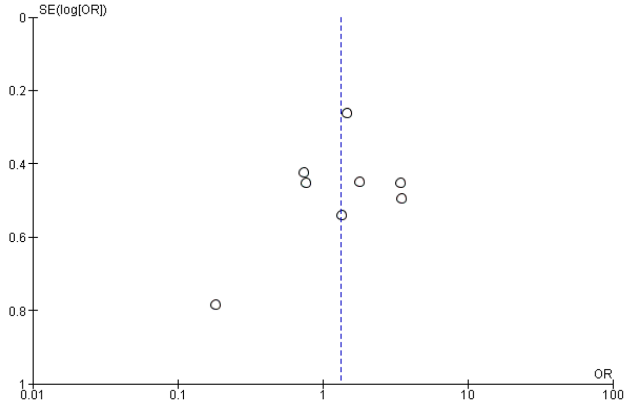


# Having a kitchen garden

| | case | | Control | | | Odds Ratio |
|---|---|---|---|---|---|---|
| Study or Subgroup | Events | Total | Events | Total | Weight | M-H, Random, 95% CI |
| Campos-Bueno 2000, 179.2 | 105 | 209 | 22 | 45 | 56.4% | 1.06 [0.55, 2.01] |
| Dowling 2000, 177.2 | 4 | 37 | 40 | 139 | 43.6% | 0.30 [0.10, 0.90] |
| **Total (95% CI)** | | **246** | | **184** | **100.0%** | **0.61 [0.18, 2.09]** |
| Total events | 109 | | 62 | | | |

Heterogeneity: Tau² = 0.59; Chi² = 3.80, df = 1 (P = 0.05); I² = 74%
Test for overall effect: Z = 0.79 (P = 0.43)

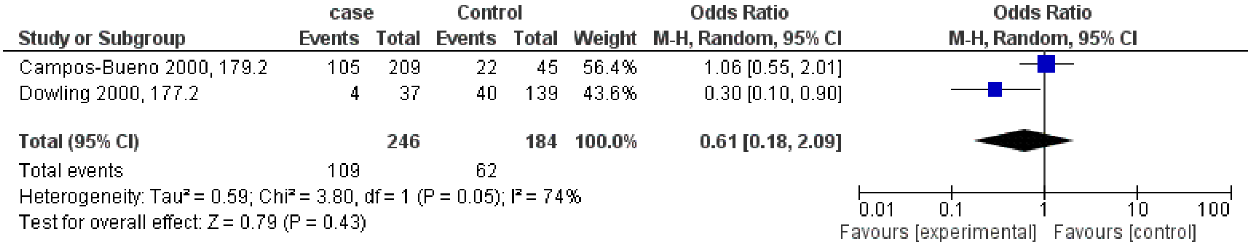


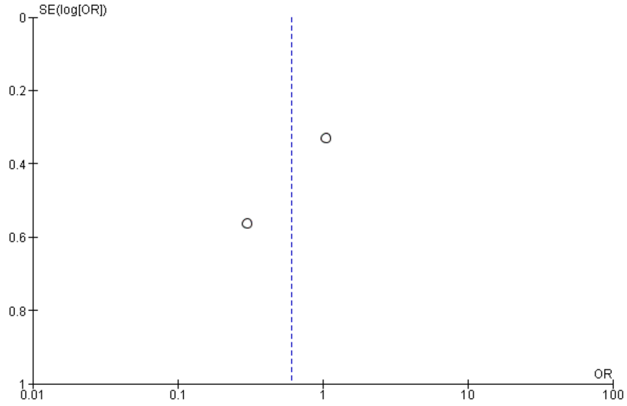

# Eating raw/unwashed vegetables

| Study or Subgroup | case Events | case Total | Control Events | Control Total | Weight | Odds Ratio M-H, Fixed, 95% CI |
|---|---|---|---|---|---|---|
| Dowling 2000, 177.1 | 40 | 155 | 4 | 21 | 27.9% | 1.48 [0.47, 4.65] |
| Dowling 2000, 177.2 | 4 | 19 | 40 | 157 | 36.4% | 0.78 [0.24, 2.49] |
| Dowling 2000, 177.3 | 0 | 10 | 44 | 166 | 28.0% | 0.13 [0.01, 2.28] |
| Larrieu 2002, 9.1 | 23 | 87 | 1 | 3 | 7.6% | 0.72 [0.06, 8.31] |
| **Total (95% CI)** | | **271** | | **347** | **100.0%** | **0.79 [0.40, 1.56]** |
| Total events | 67 | | 89 | | | |

Heterogeneity: Chi² = 2.67, df = 3 (P = 0.44); I² = 0%
Test for overall effect: Z = 0.68 (P = 0.50)

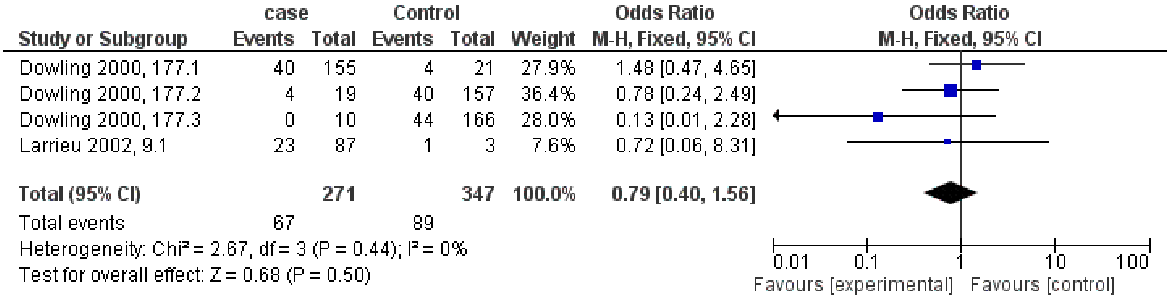


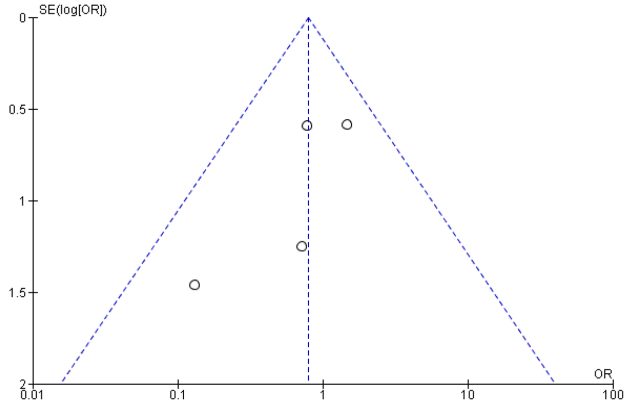


# Low income

| Study or Subgroup | case Events | case Total | Control Events | Control Total | Weight | Odds Ratio M-H, Fixed, 95% CI |
|---|---|---|---|---|---|---|
| Abou-Daoud 1964, 90.1 | 18 | 33 | 16 | 35 | 29.0% | 1.43 [0.55, 3.70] |
| Dowling 2000, 177.1 | 26 | 88 | 18 | 88 | 52.1% | 1.63 [0.82, 3.26] |
| Larrieu 2002, 9.2 | 18 | 59 | 5 | 30 | 18.9% | 2.20 [0.72, 6.65] |
| **Total (95% CI)** | | **180** | | **153** | **100.0%** | **1.68 [1.02, 2.76]** |
| Total events | 62 | | 39 | | | |

Heterogeneity: Chi² = 0.34, df = 2 (P = 0.84); I² = 0%
Test for overall effect: Z = 2.04 (P = 0.04)

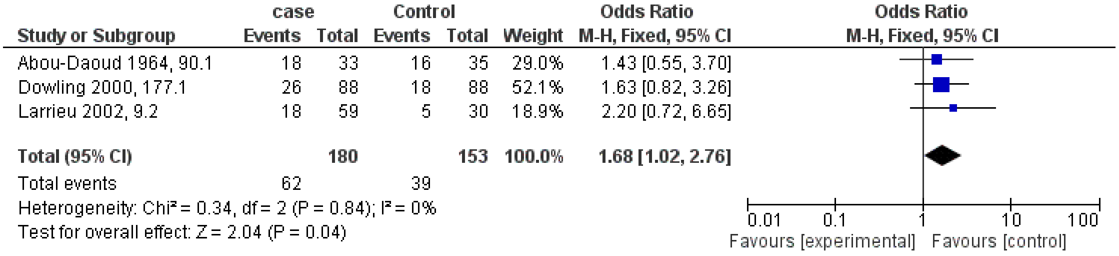


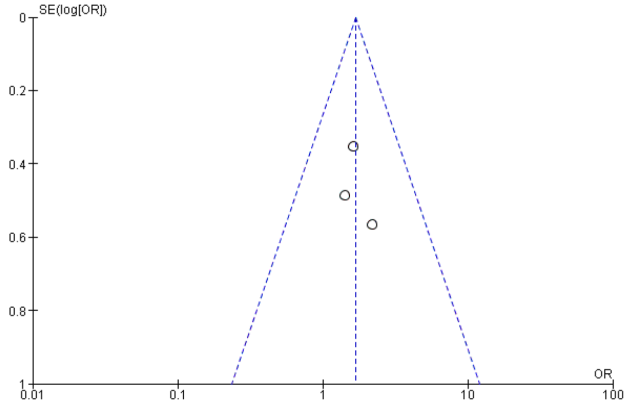

## Low education

| Study or Subgroup | case Events | case Total | Control Events | Control Total | Weight | Odds Ratio M-H, Fixed, 95% CI |
|---|---|---|---|---|---|---|
| Abou-Daoud 1964, 90.2 | 29 | 56 | 16 | 35 | 28.4% | 1.28 [0.55, 2.97] |
| Campos-Bueno 2000, 179.1 | 109 | 207 | 18 | 47 | 41.6% | 1.79 [0.94, 3.43] |
| Larrieu 2002, 9.1 | 20 | 71 | 1 | 6 | 4.0% | 1.96 [0.22, 17.85] |
| Larrieu 2002, 9.3 | 7 | 30 | 17 | 60 | 26.0% | 0.77 [0.28, 2.13] |
| **Total (95% CI)** | | **364** | | **148** | **100.0%** | **1.39 [0.89, 2.16]** |
| Total events | 165 | | 52 | | | |

Heterogeneity: Chi² = 2.02, df = 3 (P = 0.57); I² = 0%
Test for overall effect: Z = 1.44 (P = 0.15)

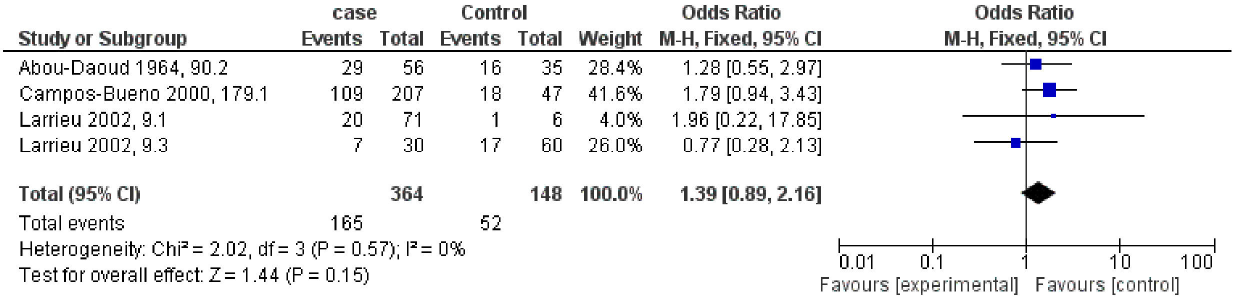


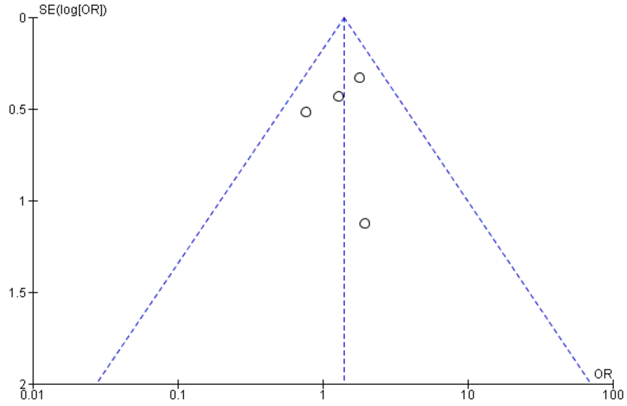


## Drinking tap or piped water

| Study or Subgroup | case Events | case Total | Control Events | Control Total | Weight | Odds Ratio M-H, Random, 95% CI |
|---|---|---|---|---|---|---|
| Dowling 2000, 177.1 | 42 | 123 | 2 | 53 | 26.0% | 13.22 [3.07, 57.00] |
| Larrieu 2002, 9.1 | 10 | 65 | 11 | 22 | 27.2% | 0.18 [0.06, 0.53] |
| Moro 2008, 128.1 | 1 | 4 | 31 | 92 | 23.0% | 0.66 [0.07, 6.57] |
| Moro 2008, 128.3 | 1 | 16 | 31 | 64 | 23.8% | 0.07 [0.01, 0.57] |
| **Total (95% CI)** | | **208** | | **231** | **100.0%** | **0.60 [0.05, 7.18]** |
| Total events | 54 | | 75 | | | |

Heterogeneity: Tau² = 5.64; Chi² = 27.92, df = 3 (P < 0.00001); I² = 89%
Test for overall effect: Z = 0.41 (P = 0.68)

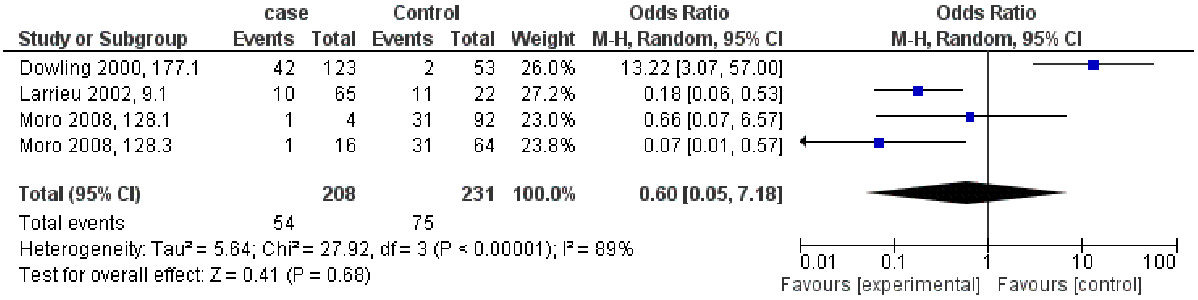


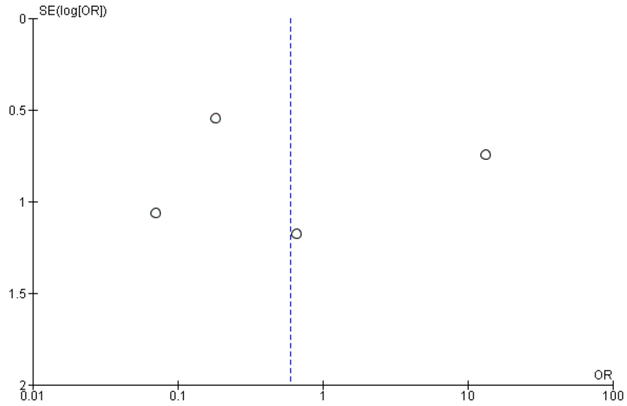

Supplement: S1 Supplementary Information — (PDF) [file pntd.0005114.s004.pdf]
