# Supplementary material for: Potential Risk Factors Associated with Human Cystic Echinococcosis: Systematic Review and Meta-analysis
Source: PLoS Negl Trop Dis. 2016 Nov 7;10(11):e0005114. doi: 10.1371/journal.pntd.0005114 (PMC5098738; doi:10.1371/journal.pntd.0005114)

CROSS-SECTIONAL studies reporting forest and funnel plot analysis on single potential risk factors.

Dog ownership

Study or Subgroup	events Events	events Total	Control Events	Control Total	Weight	Odds Ratio M-H, Random, 95% CI
Bai 2002, 217.2	8	675	1	193	2.0%	2.30 [0.29, 18.53]
Bingham 2014, 16.1	16	226	9	69	8.2%	0.51 [0.21, 1.21]
Carmona 1998, 176.1	94	5562	65	3843	18.7%	1.00 [0.73, 1.37]
Ok 2007, 164.2	2	264	7	5829	3.3%	6.35 [1.31, 30.71]
Shambesh 1999, 96.1	210	9709	129	11141	20.9%	1.89 [1.51, 2.35]
Tiaoying 2005, 54.1	185	2482	22	496	15.5%	1.74 [1.10, 2.73]
Wang 2001, 33.6	35	1818	29	1792	14.5%	1.19 [0.73, 1.96]
Yang 2006, 15.1	44	2055	64	4020	17.0%	1.35 [0.92, 1.99]
**Total (95% CI)**		**22791**		**27383**	**100.0%**	**1.37 [1.01, 1.86]**
Total events	594		326			

Heterogeneity: $Tau^2 = 0.10$; $Chi^2 = 21.19$, df = 7 (P = 0.003); $I^2 = 67\%$
Test for overall effect: Z = 2.02 (P = 0.04)

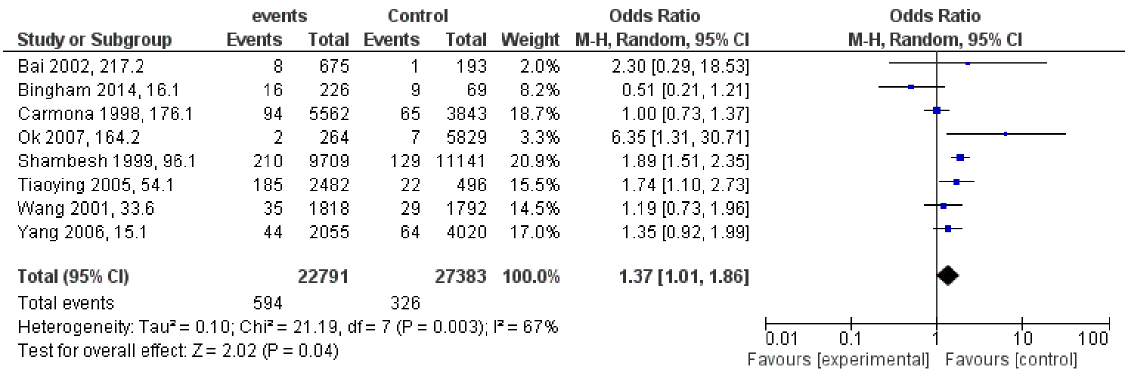

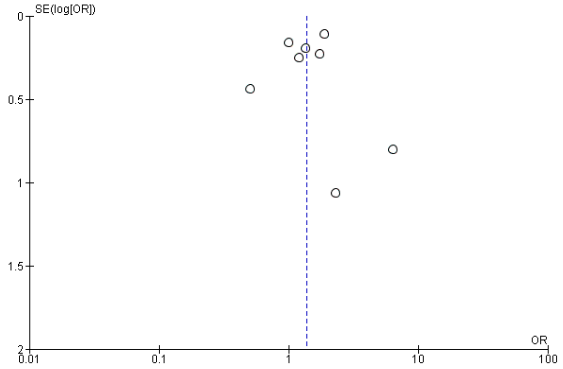

Feeding dogs with viscera

Study or Subgroup	events Events	events Total	Control Events	Control Total	Weight	Odds Ratio M-H, Fixed, 95% CI
Bingham 2014, 16.3	5	70	10	137	33.9%	0.98 [0.32, 2.98]
Carmona 1998, 176.3	11	354	80	5153	53.8%	2.03 [1.07, 3.86]
Ok 2007, 164.1	1	864	8	5229	12.2%	0.76 [0.09, 6.05]
**Total (95% CI)**		**1288**		**10519**	**100.0%**	**1.52 [0.88, 2.62]**
Total events	17		98			

Heterogeneity: $Chi^2 = 1.83$, df = 2 (P = 0.40); $I^2 = 0\%$
Test for overall effect: Z = 1.50 (P = 0.13)

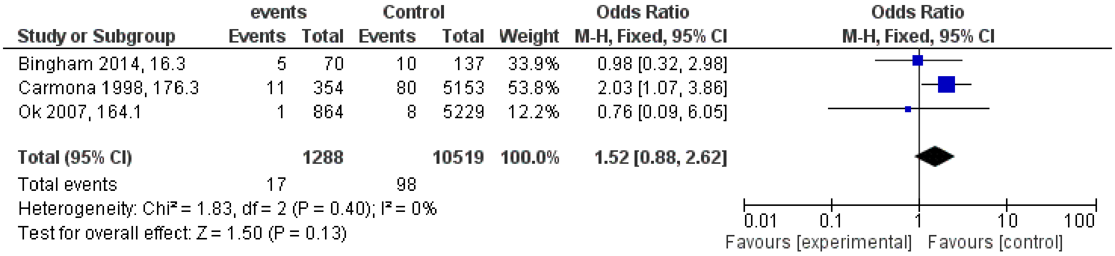

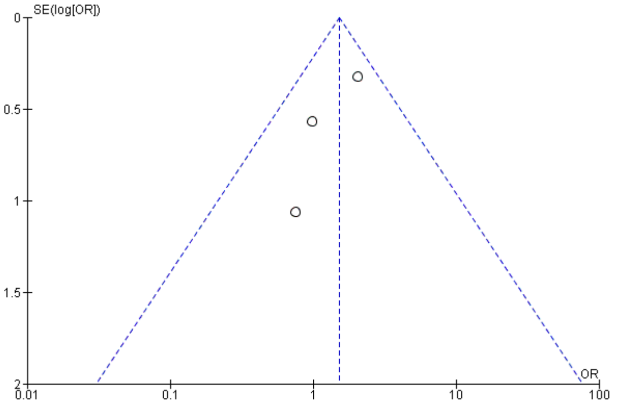

Slaughter at home

Study or Subgroup	events Events	events Total	Control Events	Control Total	Weight	Odds Ratio M-H, Fixed, 95% CI
Bingham 2014, 16.1	12	163	14	144	10.9%	0.74 [0.33, 1.65]
Carmona 1998, 176.1	49	2529	103	6867	42.9%	1.30 [0.92, 1.83]
Wang 2001, 33.6	51	2348	15	1277	15.0%	1.87 [1.05, 3.34]
Yang 2006, 15.1	35	2393	40	2380	31.2%	0.87 [0.55, 1.37]
**Total (95% CI)**		**7433**		**10668**	**100.0%**	**1.19 [0.94, 1.50]**
Total events	147		172			

Heterogeneity: Chi² = 5.74, df = 3 (P = 0.12); I² = 48%
Test for overall effect: Z = 1.44 (P = 0.15)

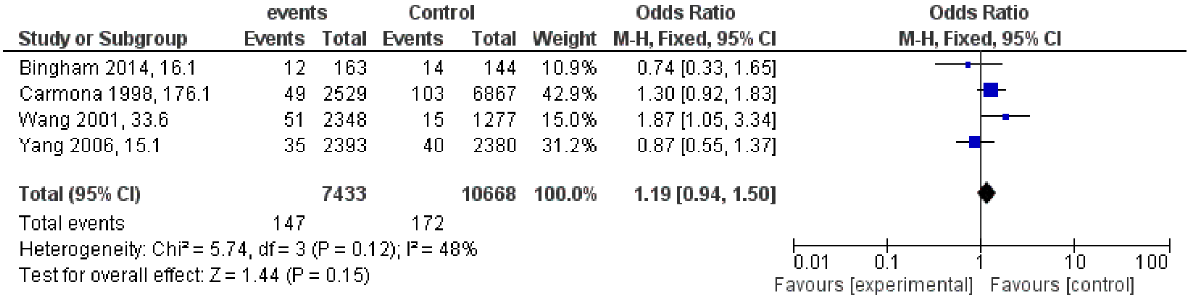

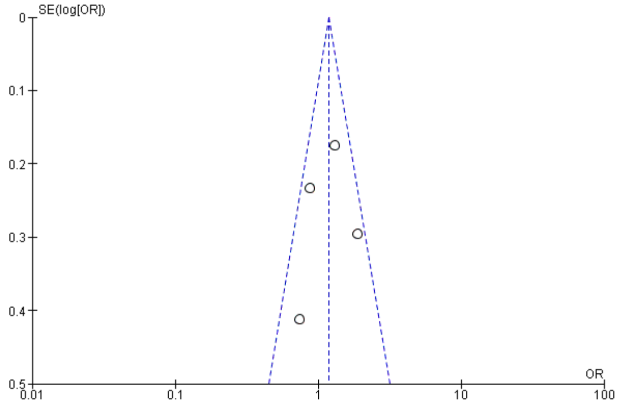

Being female

Study or Subgroup	events Events	events Total	Control Events	Control Total	Weight	Odds Ratio M-H, Random, 95% CI
Bchir 1991, 68	34	793	16	641	11.4%	1.75 [0.96, 3.20]
Bingham 2014, 16.1	22	263	11	77	8.3%	0.55 [0.25, 1.19]
Carmona 1998, 176.1	86	5076	71	4431	19.9%	1.06 [0.77, 1.45]
Shambesh 1999, 96.1	211	10356	128	9864	23.4%	1.58 [1.27, 1.97]
Stewart 2013, 31	4	129	0	481	0.8%	34.53 [1.85, 645.59]
Tiaoying 2005, 54.1	134	1660	82	1539	21.1%	1.56 [1.17, 2.07]
Yang 2006, 15.1	44	2268	31	2505	15.1%	1.58 [0.99, 2.51]
**Total (95% CI)**		**20545**		**19538**	**100.0%**	**1.38 [1.06, 1.80]**
Total events	535		339			

Heterogeneity: Tau² = 0.07; Chi² = 15.71, df = 6 (P = 0.02); I² = 62%
Test for overall effect: Z = 2.39 (P = 0.02)

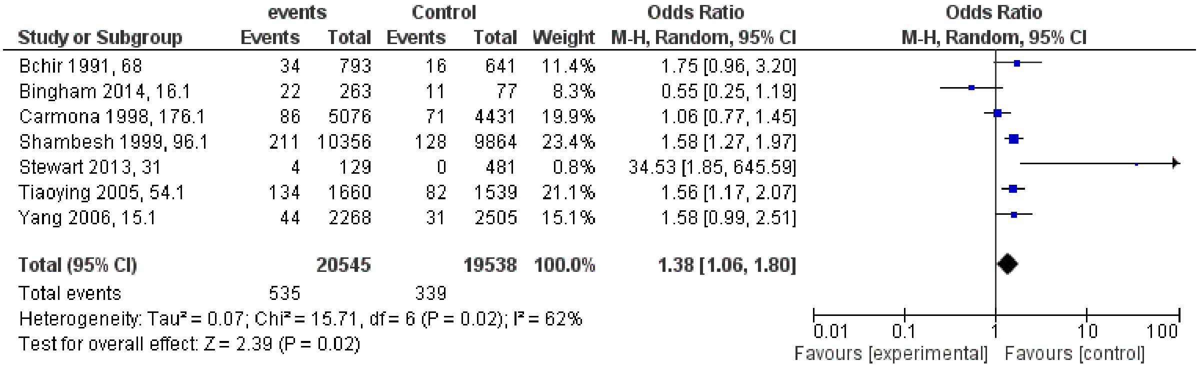

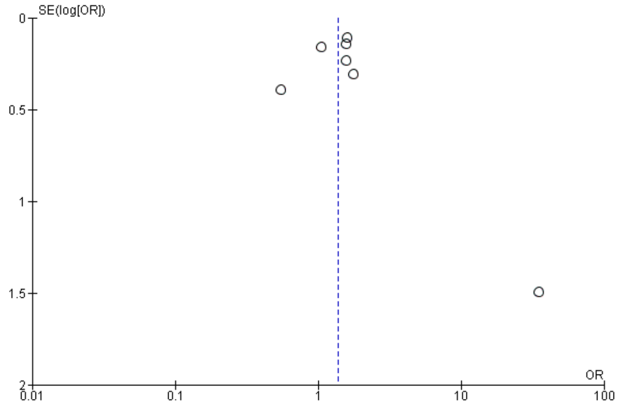

Age over 16

Study or Subgroup	events Events	events Total	Control Events	Control Total	Weight	Odds Ratio M-H, Fixed, 95% CI
Bai 2002, 217.2	7	460	2	422	6.7%	3.25 [0.67, 15.71]
Bingham 2014, 16.1	33	330	3	189	11.1%	6.89 [2.08, 22.78]
Carmona 1998, 176.1	136	5864	20	3617	78.2%	4.27 [2.67, 6.84]
Yang 2006, 15.1	74	3023	1	1750	4.0%	43.89 [6.10, 315.98]
**Total (95% CI)**		**9677**		**5978**	**100.0%**	**6.08 [4.05, 9.13]**
Total events	250		26			

Heterogeneity: Chi² = 6.66, df = 3 (P = 0.08); I² = 55%

Test for overall effect: Z = 8.69 (P < 0.00001)

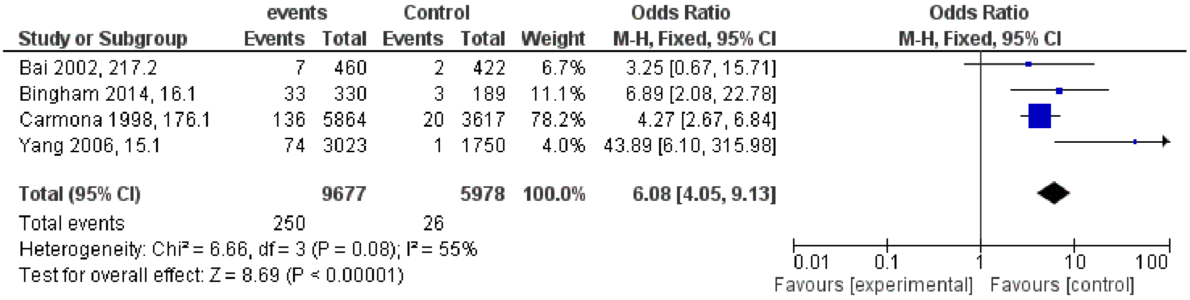

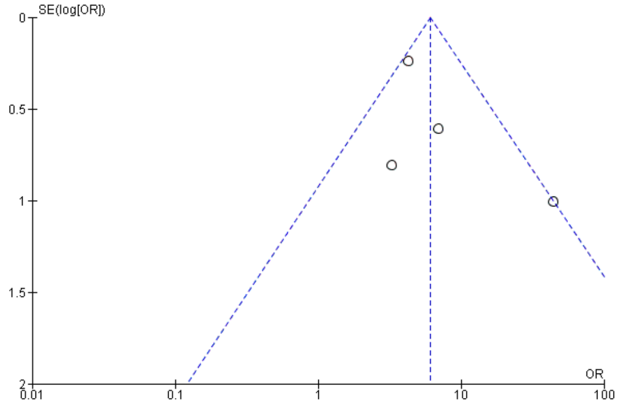

Ethnic group: Han

Study or Subgroup	events Events	events Total	Control Events	Control Total	Weight	Odds Ratio M-H, Random, 95% CI
Wang 2001, 33.3	4	81	45	1763	23.9%	1.98 [0.70, 5.65]
Wang 2001, 33.5	5	417	12	1444	23.9%	1.45 [0.51, 4.13]
Wang 2005, 110	49	1754	4	1585	24.1%	11.36 [4.09, 31.55]
Yang 2006, 15.1	35	2447	40	2326	28.1%	0.83 [0.52, 1.31]
**Total (95% CI)**		**4699**		**7118**	**100.0%**	**2.19 [0.66, 7.26]**
Total events	93		101			

Heterogeneity: Tau² = 1.27; Chi² = 23.51, df = 3 (P < 0.0001); I² = 87%

Test for overall effect: Z = 1.29 (P = 0.20)

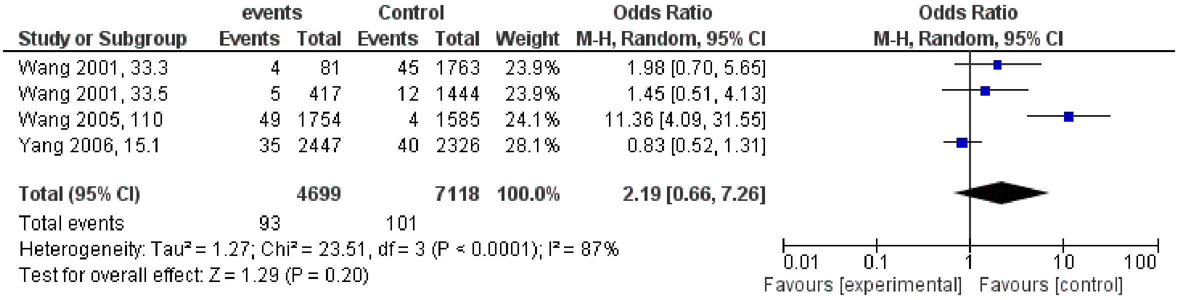

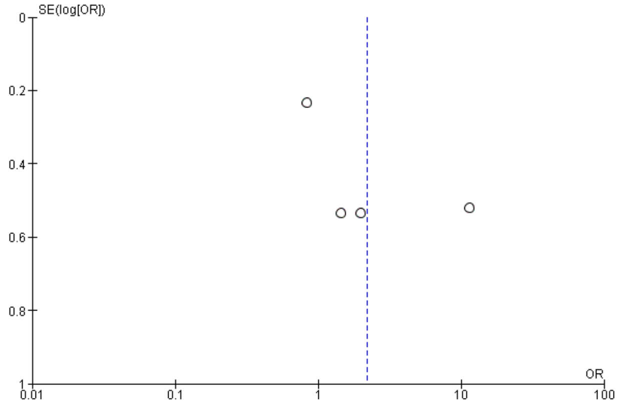

Living in rural areas

Study or Subgroup	events Events	events Total	Control Events	Control Total	Weight	Odds Ratio M-H, Fixed, 95% CI
Bingham 2014, 16.1	25	221	32	398	83.1%	1.46 [0.84, 2.53]
Ok 2007, 164.1	5	1853	4	4240	10.0%	2.87 [0.77, 10.68]
Yang 2006, 15.1	69	3139	1	490	6.9%	10.99 [1.52, 79.32]
**Total (95% CI)**		**5213**		**5128**	**100.0%**	**2.26 [1.41, 3.61]**
Total events	99		37			

Heterogeneity: Chi² = 5.01, df = 2 (P = 0.08); I² = 60%

Test for overall effect: Z = 3.41 (P = 0.0007)

Odds Ratio
M-H, Fixed, 95% CI

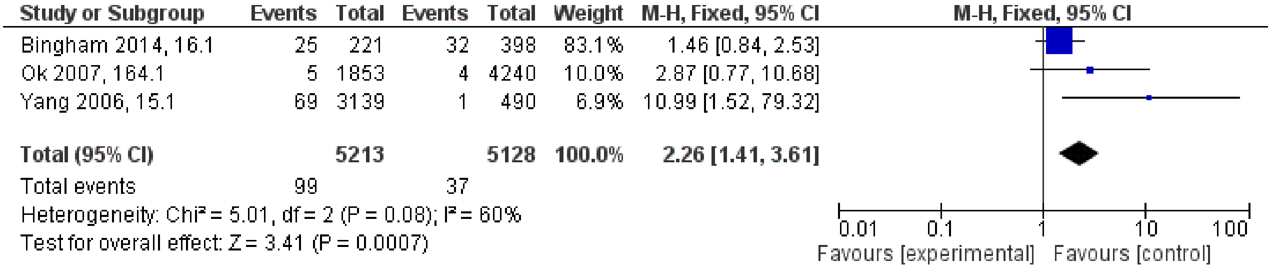

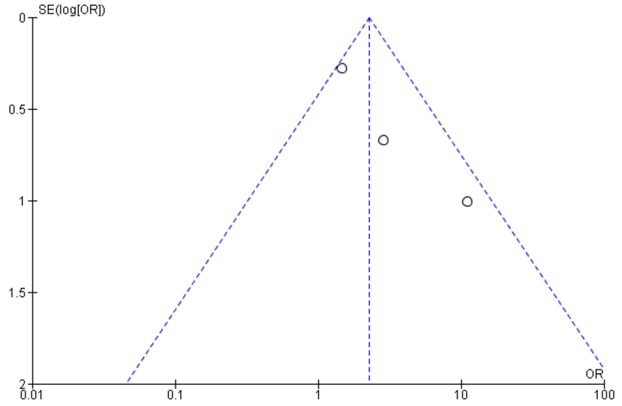

Family history of CE

Study or Subgroup	events Events	events Total	Control Events	Control Total	Weight	Odds Ratio M-H, Fixed, 95% CI
Bingham 2014, 16.1	10	79	21	249	15.4%	1.57 [0.71, 3.50]
Carmona 1998, 176.1	37	1964	118	7431	84.6%	1.19 [0.82, 1.73]
**Total (95% CI)**		**2043**		**7680**	**100.0%**	**1.25 [0.89, 1.75]**
Total events	47		139			

Heterogeneity: Chi² = 0.39, df = 1 (P = 0.53); I² = 0%

Test for overall effect: Z = 1.29 (P = 0.20)

Odds Ratio
M-H, Fixed, 95% CI

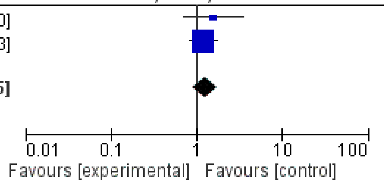

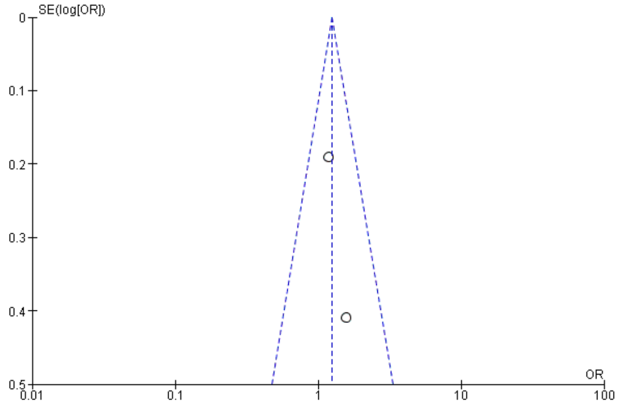

Being a farmer

Study or Subgroup	events Events	events Total	Control Events	Control Total	Weight	Odds Ratio M-H, Random, 95% CI
Shambesh 1999, 96.3	14	544	1003	60116	45.4%	1.56 [0.91, 2.66]
Tiaoying 2005, 54.2	0	35	46	1347	13.4%	0.39 [0.02, 6.53]
Yang 2006, 15.1	68	3068	7	1705	41.2%	5.50 [2.52, 12.00]
**Total (95% CI)**		**3647**		**63168**	**100.0%**	**2.18 [0.66, 7.22]**
Total events	82		1056			

Heterogeneity: Tau² = 0.75; Chi² = 8.99, df = 2 (P = 0.01); I² = 78%
Test for overall effect: Z = 1.27 (P = 0.20)

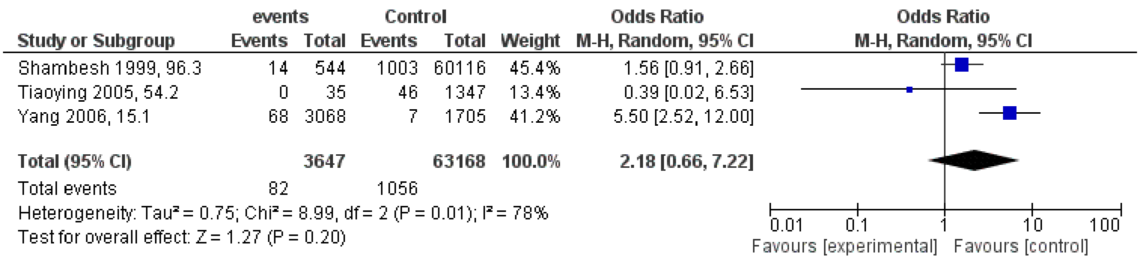

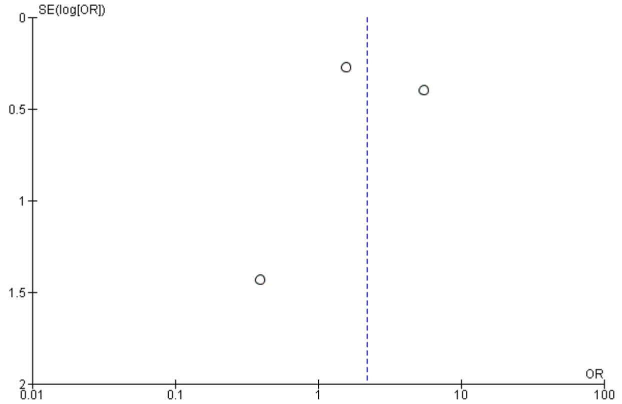

Livestock owner

Study or Subgroup	events Events	events Total	Control Events	Control Total	Weight	Odds Ratio M-H, Fixed, 95% CI
Bingham 2014, 16.1	9	130	17	183	20.1%	0.73 [0.31, 1.68]
Ok 2007, 164.1	2	782	7	5311	2.7%	1.94 [0.40, 9.37]
Ok 2007, 164.2	1	526	8	5567	2.1%	1.32 [0.17, 10.60]
Wang 2001, 33.6	39	2062	24	1487	41.8%	1.18 [0.70, 1.96]
Yang 2006, 15.2	62	4069	13	704	33.3%	0.82 [0.45, 1.50]
**Total (95% CI)**		**7569**		**13252**	**100.0%**	**0.99 [0.70, 1.40]**
Total events	113		69			

Heterogeneity: Chi² = 2.09, df = 4 (P = 0.72); I² = 0%
Test for overall effect: Z = 0.05 (P = 0.96)

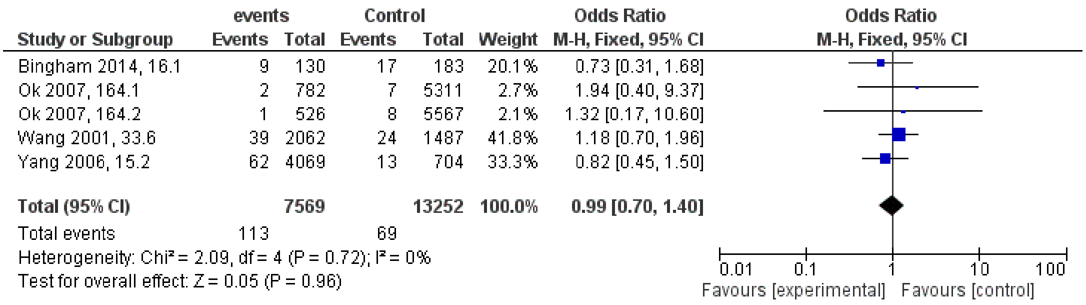

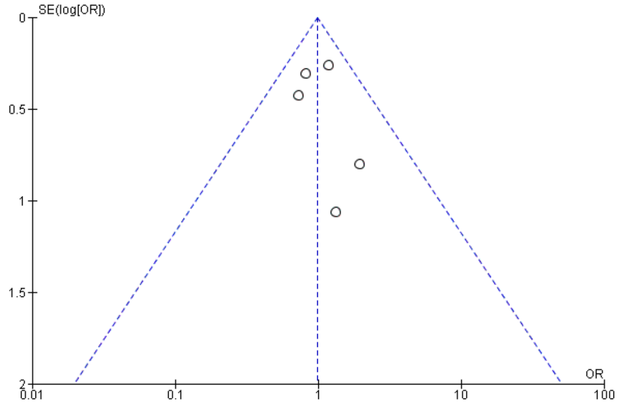

Eating raw/unwashed vegetables

Study or Subgroup	events Events	events Total	Control Events	Control Total	Weight	Odds Ratio M-H, Fixed, 95% CI
Ok 2007, 164.2	5	2017	4	4076	12.4%	2.53 [0.68, 9.43]
Yang 2006, 15.1	64	4112	11	661	87.6%	0.93 [0.49, 1.78]
**Total (95% CI)**		**6129**		**4737**	**100.0%**	**1.13 [0.63, 2.05]**
Total events	69		15			

Heterogeneity: Chi² = 1.78, df = 1 (P = 0.18); I² = 44%
Test for overall effect: Z = 0.41 (P = 0.68)

Odds Ratio
M-H, Fixed, 95% CI

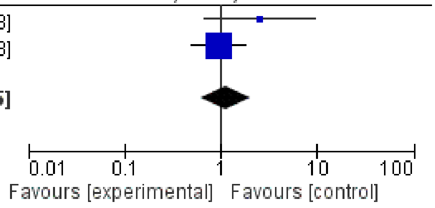

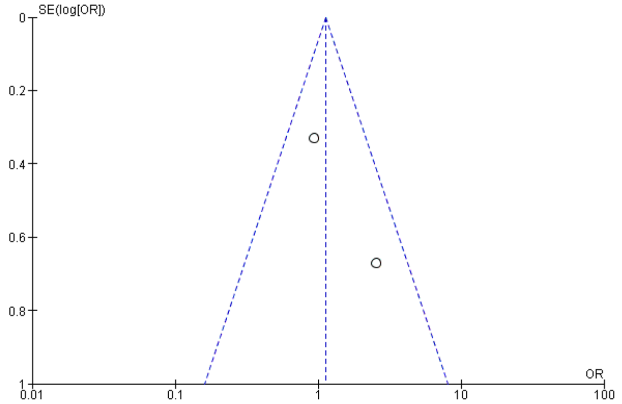

Low education

Study or Subgroup	events Events	events Total	Control Events	Control Total	Weight	Odds Ratio M-H, Random, 95% CI
Bai 2002, 217.2	4	409	5	473	53.6%	0.92 [0.25, 3.47]
Bingham 2014, 16.1	30	233	1	87	46.4%	12.71 [1.71, 94.69]
**Total (95% CI)**		**642**		**560**	**100.0%**	**3.12 [0.19, 51.32]**
Total events	34		6			

Heterogeneity: Tau² = 3.35; Chi² = 5.46, df = 1 (P = 0.02); I² = 82%
Test for overall effect: Z = 0.80 (P = 0.43)

Odds Ratio
M-H, Random, 95% CI

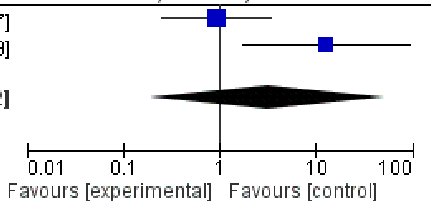

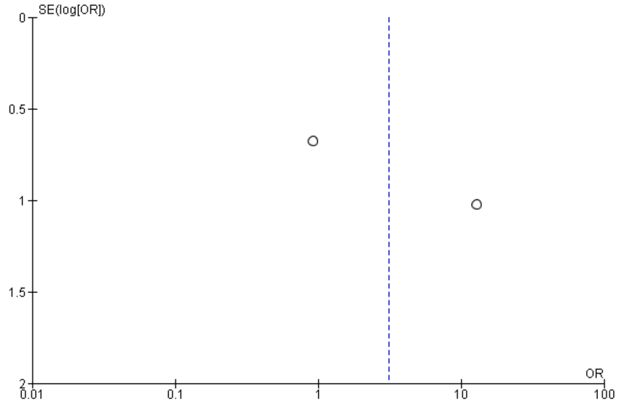

Low income

Study or Subgroup	events Events	events Total	Control Events	Control Total	Weight	Odds Ratio M-H, Fixed, 95% CI
Bingham 2014, 16.2	24	201	8	120	65.7%	1.90 [0.82, 4.37]
Ok 2007, 164.1	2	2005	7	4088	34.3%	0.58 [0.12, 2.80]
**Total (95% CI)**		**2206**		**4208**	**100.0%**	**1.45 [0.72, 2.91]**
Total events	26		15			

Heterogeneity: Chi² = 1.69, df = 1 (P = 0.19); I² = 41%

Test for overall effect: Z = 1.04 (P = 0.30)

Odds Ratio M-H, Fixed, 95% CI

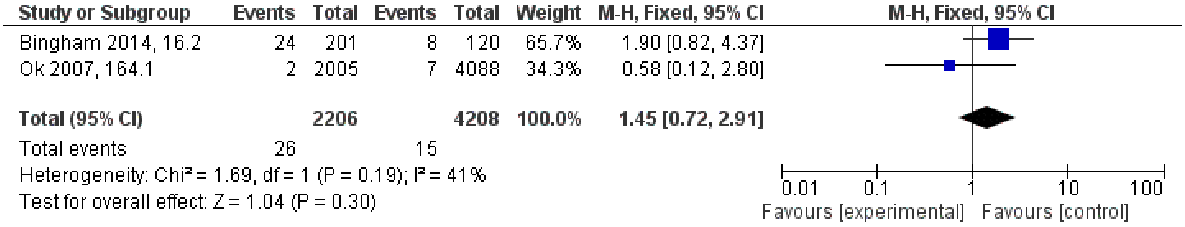

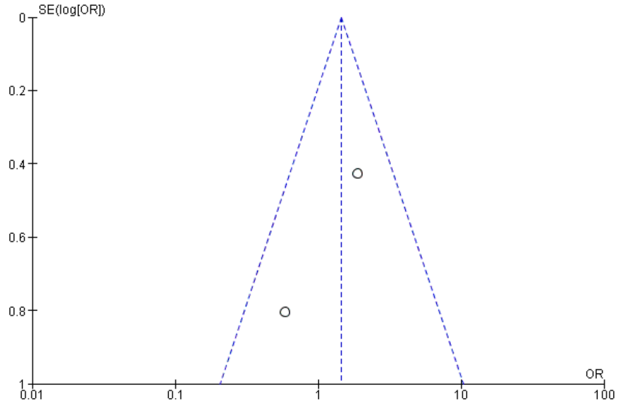

No knowledge on *Echinococcus*

Study or Subgroup	events Events	events Total	Control Events	Control Total	Weight	Odds Ratio M-H, Random, 95% CI
Wang 2001, 33.6	22	1797	44	1838	50.3%	0.51 [0.30, 0.85]
Yang 2006, 15.1	15	3297	60	1476	49.7%	0.11 [0.06, 0.19]
**Total (95% CI)**		**5094**		**3314**	**100.0%**	**0.23 [0.05, 1.07]**
Total events	37		104			

Heterogeneity: Tau² = 1.12; Chi² = 15.55, df = 1 (P < 0.0001); I² = 94%

Test for overall effect: Z = 1.88 (P = 0.06)

Odds Ratio M-H, Random, 95% CI

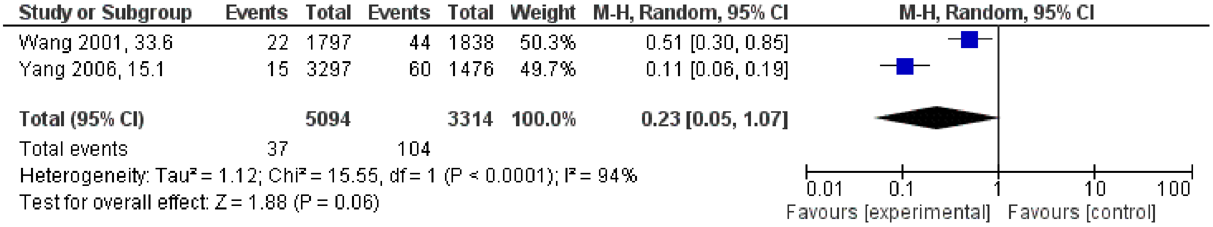

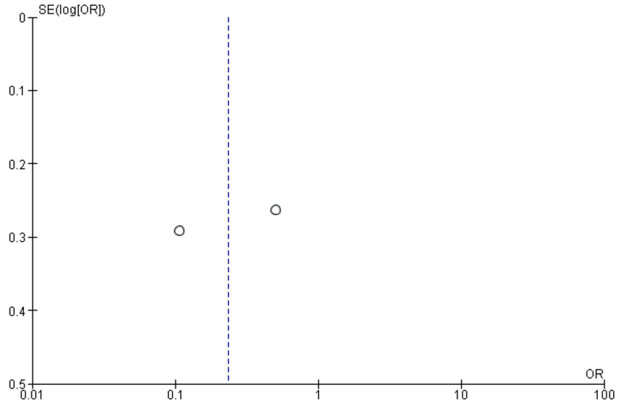

Drinking unboiled water

Study or Subgroup	events Events	events Total	Control Events	Control Total	Weight	Odds Ratio M-H, Fixed, 95% CI
Carmona 1998, 176.1	105	6601	44	2616	60.2%	0.94 [0.66, 1.35]
Yang 2006, 15.1	47	3549	28	1224	39.8%	0.57 [0.36, 0.92]
**Total (95% CI)**		**10150**		**3840**	**100.0%**	**0.80 [0.60, 1.06]**
Total events	152		72			

Heterogeneity: Chi² = 2.75, df = 1 (P = 0.10); I² = 64%

Test for overall effect: Z = 1.58 (P = 0.12)

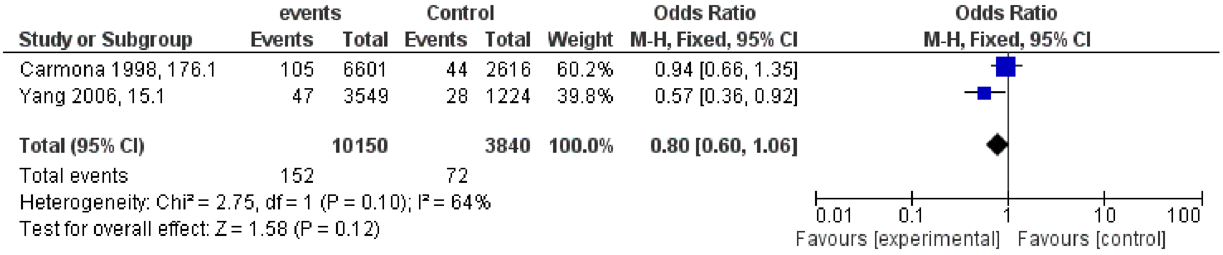

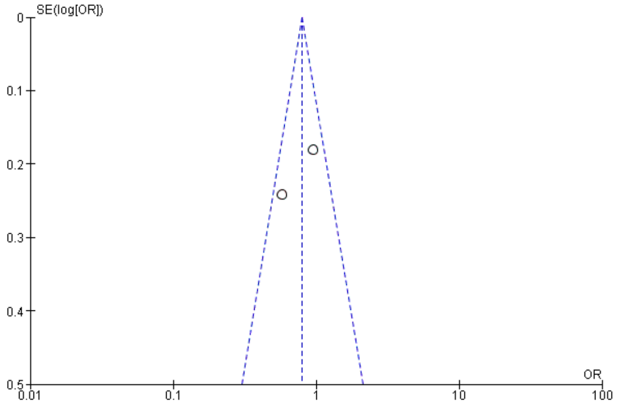

Drinking tap or piped water

Study or Subgroup	events Events	events Total	Control Events	Control Total	Weight	Odds Ratio M-H, Fixed, 95% CI
Bingham 2014, 16.1	28	287	2	36	12.1%	1.84 [0.42, 8.06]
Ok 2007, 164.1	7	4796	11	7390	32.7%	0.98 [0.38, 2.53]
Yang 2006, 15.2	8	546	63	4099	55.2%	0.95 [0.45, 2.00]
**Total (95% CI)**		**5629**		**11525**	**100.0%**	**1.07 [0.63, 1.81]**
Total events	43		76			

Heterogeneity: Chi² = 0.64, df = 2 (P = 0.73); I² = 0%

Test for overall effect: Z = 0.25 (P = 0.80)

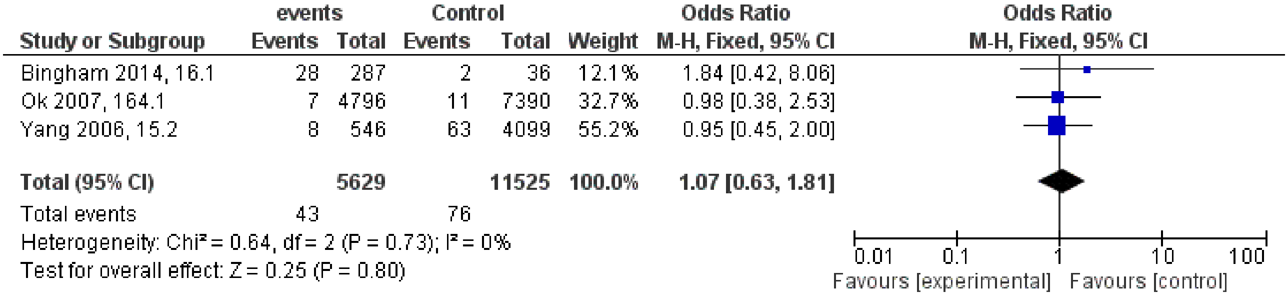

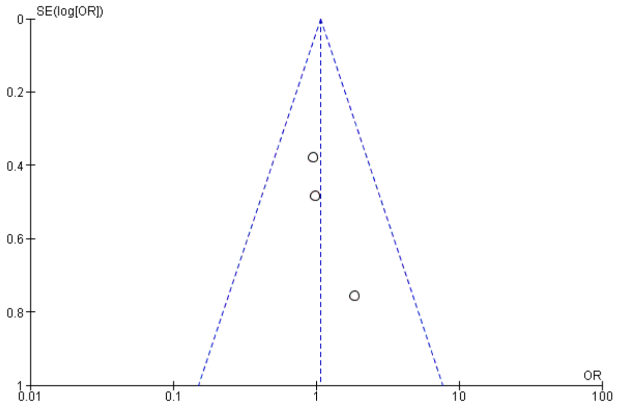

Drinking well water

	events		Control			Odds Ratio
Study or Subgroup	Events	Total	Events	Total	Weight	M-H, Fixed, 95% CI
Bingham 2014, 16.2	1	24	23	293	7.4%	0.51 [0.07, 3.95]
Ok 2007, 164.3	0	209	18	11977	1.4%	1.54 [0.09, 25.69]
Yang 2006, 15.3	37	2867	34	1778	91.2%	0.67 [0.42, 1.07]
**Total (95% CI)**		**3100**		**14048**	**100.0%**	**0.67 [0.43, 1.06]**
Total events	38		75			

Heterogeneity: Chi² = 0.41, df = 2 (P = 0.82); I² = 0%
Test for overall effect: Z = 1.73 (P = 0.08)

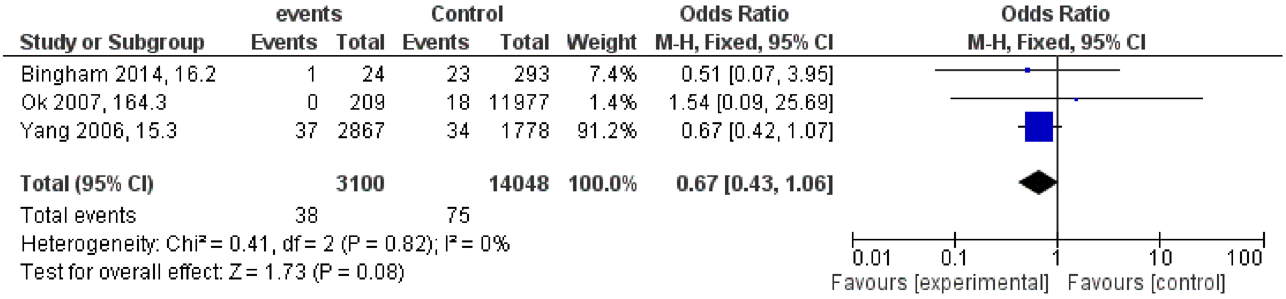

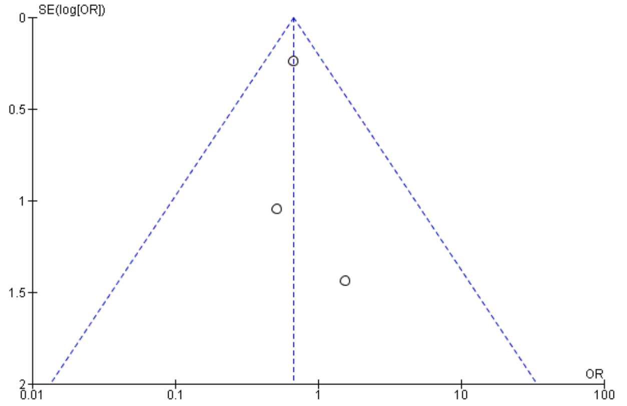

Drinking spring water

	events		Control			Odds Ratio
Study or Subgroup	Events	Total	Events	Total	Weight	M-H, Fixed, 95% CI
Bingham 2014, 16.3	1	12	11	293	3.3%	2.33 [0.28, 19.69]
Yang 2006, 15.4	23	1138	48	3507	96.7%	1.49 [0.90, 2.45]
**Total (95% CI)**		**1150**		**3800**	**100.0%**	**1.51 [0.93, 2.47]**
Total events	24		59			

Heterogeneity: Chi² = 0.16, df = 1 (P = 0.69); I² = 0%
Test for overall effect: Z = 1.66 (P = 0.10)

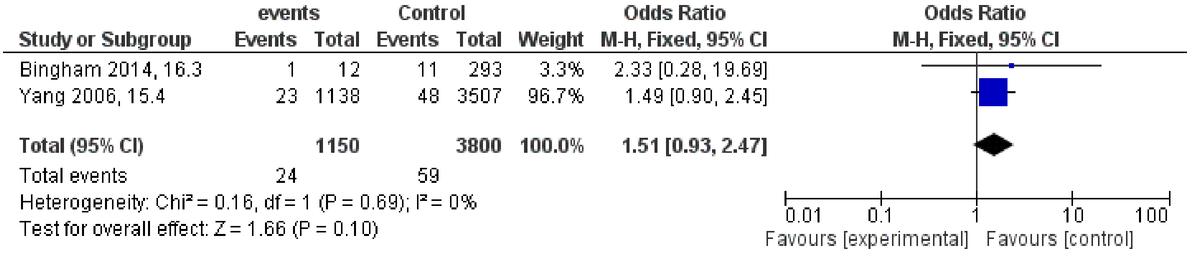

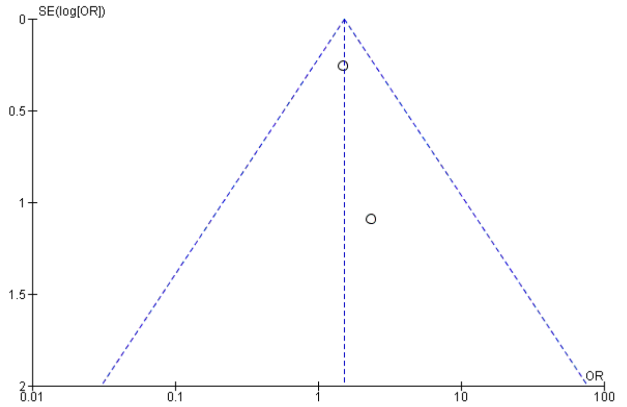

Supplement: S2 Supplementary Information — (PDF) [file pntd.0005114.s005.pdf]
