# Supplementary material for: Potential Risk Factors Associated with Human Cystic Echinococcosis: Systematic Review and Meta-analysis
Source: PLoS Negl Trop Dis. 2016 Nov 7;10(11):e0005114. doi: 10.1371/journal.pntd.0005114 (PMC5098738; doi:10.1371/journal.pntd.0005114)
Supplement: S1 Flow Diagram — (DOC) [file pntd.0005114.s006.doc]

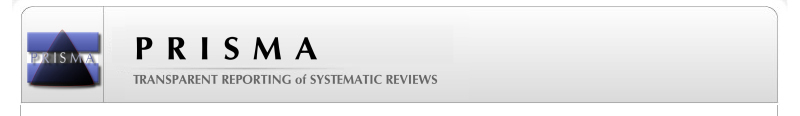
**PRISMA 2009 Flow Diagram**

**Screening**

**Included**

**Eligibility**

**Identification**

Records identified through database searching
(n = 1,367)

Additional records identified through other sources
(n = 0 )

Records after duplicates removed
(n = 1,312)

Records screened
(n = 251 )

Records excluded
(n = 1061 )

Full-text articles assessed for eligibility
(n = 251 )

Full-text articles excluded, with reasons
(n = 212 )

Studies included in qualitative synthesis
(n = 39 )

Studies included in quantitative synthesis (meta-analysis)
(n = 21 )
